# The physical map of wheat chromosome 1BS provides insights into its gene space organization and evolution

**DOI:** 10.1186/gb-2013-14-12-r138

**Published:** 2013-12-20

**Authors:** Dina Raats, Zeev Frenkel, Tamar Krugman, Itay Dodek, Hanan Sela, Hana Šimková, Federica Magni, Federica Cattonaro, Sonia Vautrin, Hélène Bergès, Thomas Wicker, Beat Keller, Philippe Leroy, Romain Philippe, Etienne Paux, Jaroslav Doležel, Catherine Feuillet, Abraham Korol, Tzion Fahima

**Affiliations:** 1 Institute of Evolution and Department of Evolutionary and Environmental Biology, University of Haifa, Haifa, Israel; 2 The Institute for Cereal Crops Improvement, Tel-Aviv University, Tel Aviv, Israel; 3 Haná Regional Center for Biotechnological and Agricultural Research, Institute of Experimental Botany, Olomouc, Czech Republic; 4 Instituto di Genomica Applicata, Udine, Italy; 5 INRA-CNRGV, Plant Genomic Resource Center, Castanet Tolosan, France; 6 Institute of Plant Biology, University of Zurich, Zurich, Switzerland; 7 INRA Genetics, Diversity and Ecophysiology of Cereals, Clermont-Ferrand, France

## Abstract

**Background:**

The wheat genome sequence is an essential tool for advanced genomic research and improvements. The generation of a high-quality wheat genome sequence is challenging due to its complex 17 Gb polyploid genome. To overcome these difficulties, sequencing through the construction of BAC-based physical maps of individual chromosomes is employed by the wheat genomics community. Here, we present the construction of the first comprehensive physical map of chromosome 1BS, and illustrate its unique gene space organization and evolution.

**Results:**

Fingerprinted BAC clones were assembled into 57 long scaffolds, anchored and ordered with 2,438 markers, covering 83% of chromosome 1BS. The BAC-based chromosome 1BS physical map and gene order of the orthologous regions of model grass species were consistent, providing strong support for the reliability of the chromosome 1BS assembly. The gene space for chromosome 1BS spans the entire length of the chromosome arm, with 76% of the genes organized in small gene islands, accompanied by a two-fold increase in gene density from the centromere to the telomere.

**Conclusions:**

This study provides new evidence on common and chromosome-specific features in the organization and evolution of the wheat genome, including a non-uniform distribution of gene density along the centromere-telomere axis, abundance of non-syntenic genes, the degree of colinearity with other grass genomes and a non-uniform size expansion along the centromere-telomere axis compared with other model cereal genomes. The high-quality physical map constructed in this study provides a solid basis for the assembly of a reference sequence of chromosome 1BS and for breeding applications.

## Background

Wheat (*Triticum aestivum* L; Poaceae) provides nearly 20% of the world’s daily food supply, as measured by calorie intake [1]. The wheat genome sequence is an essential tool for advanced genomic research and improvements that will enable growers to meet the increasing demands for high-quality food and feed produced in an environmentally sensitive, sustainable and profitable manner. The generation of a high-quality reference genome sequence for bread wheat is a challenge since it has a complex hexaploid (2n = 6x = 42) genome, 17 Gb in size, which consists of more than 80% repetitive elements [2]. The polyploidy and the repetitive nature of the wheat genome are barriers to the high-quality assembly of whole genome shotgun sequences. The International Wheat Genome Sequencing Consortium (IWGSC) [3], established with the goal of fully sequencing the wheat genome [4], has adopted a chromosome-by-chromosome sequencing strategy with the construction of bacterial artificial chromosome (BAC)-based physical maps as a necessary intermediate step. The current study is part of the European TriticeaeGenome FP7 project [5], which was established to construct physical maps of group 1 and 3 chromosomes of wheat and barley [6].

The ability to sort out individual wheat chromosomes by flow cytometry has been key to the success in reducing the complexity of wheat genome analysis. This approach has made it possible to develop chromosome-specific BAC libraries using aneuploid lines of the model wheat genotype Chinese Spring (CS) [7-9]. Nowadays, chromosome and chromosome-arm-specific BAC libraries are available for all wheat chromosomes [9]. Physical maps of individual wheat chromosomes and chromosome arms can be established via various fingerprinting technologies (for example, [10,11]). BAC clones are typically digested with restriction enzymes, which produce a specific pattern of DNA fragments (referred to as bands hereafter) for each BAC clone. These DNA patterns (referred to as fingerprints) are then used to find significant overlaps between BAC clones and to arrange them into physical BAC contigs. In the current study we have used the high-information-content fingerprinting (HICF) method, which produces particularly diverse fingerprint patterns [10]. FingerPrinted Contigs (FPC) [12] is a contig assembly program, which has been used in several grass genome projects (for example, [13-15]). However, the application of FPC is not sufficiently effective for analysis of complex genomes, such as wheat and barley. Due to the high level of repetitive DNA in the wheat genome, the criteria that FPC uses for BAC contig assembly often result in very short contigs and unreliable assembly. Moreover, the presence of repetitive or poorly fingerprinted ‘questionable’ clones (Q-clones) can lead to false overlaps and thus wrongly assembled contigs.

Linear Topological Contig (LTC) is a novel analytical program for contig assembly, which was developed by Frenkel *et al*. [16] and is available from the Institute of Evolution website [17], University of Haifa. LTC has several major advantages over the standard FPC software [12], which are crucial for the assembly of complex genomes, such as wheat. The algorithms implemented in LTC facilitate the construction of longer and more reliable BAC-contigs, compared to FPC. LTC provides a visual representation of significant clone overlaps as a network of parallel clone connections, thus allowing straightforward identification of Q-clones and Q-overlaps (overlaps that are not confirmed by multiple paralleled connections). LTC improves contig assembly by breaking branching contigs into linear structures that do not contradict the one-dimensional linear organization of the chromosome. An end-to-end merging strategy can then be applied to combine linear contigs into long scaffolds. This strategy can reveal the correct orientation and order of contigs within scaffolds along the chromosome, and thereby reduce anchoring efforts. In the current study, a physical map for 1BS was constructed using the LTC program and the BAC contig assemblies produced by LTC and FPC were compared.

Once a physical map has been assembled, the BAC contigs are usually anchored with molecular markers to high-density genetic maps. In wheat, however, recombination-based mapping cannot provide resolution necessary for the anchoring, due to the uneven distribution of crossing-over events along the chromosomes and the strong long-distance suppressing effect of the centromeres on recombination [18-21]. Cytogenetic stocks in wheat (for example, ditelosomic lines and chromosome deletion lines) have been used to map and anchor BAC contigs [22]. However, mapping using these stocks is limited due to large deletion sizes (the average deletion bin is approximately 35 Mb) and the inability to order markers within a given bin [22]. The compilation of synteny information from the completely sequenced genomes of model grasses such as rice (*Oryza sativa*) [14], sorghum (*Sorghum bicolor*) [15] and *Brachypodium* (*Brachypodium distachyon*) [13] has made it possible to deduce a hypothetical gene content and order in the syntenic regions of grass species for which no reference genome is available such as wheat and barley [23-26]. This comparative genomic strategy, referred to as the GenomeZipper approach, has been shown to be efficient in the BAC-based physical mapping of the barley genome [27].

The estimated length of the short arm of wheat chromosome 1B (1BS) is approximately 314 Mb [8,28], which is larger than the complete *Brachypodium* genome (270 Mb) and is 1.9% of the entire hexaploid wheat genome. This chromosome arm carries various disease and pest-resistance genes, and storage protein genes that directly affect grain quality [19,29], as well as many other economically important genes [30].

Here we present the final assembly and analysis of the physical map of the 1BS chromosome of bread wheat, with comprehensive corresponding genomic resources including: (1) a deep-coverage BAC library; (2) a high-information-content BAC fingerprint data set; (3) assembled BAC contigs subsequently elongated to scaffolds using LTC; (4) a transcriptional map based on the wheat gene microarray containing approximately 40 K NCBI UniGene expressed sequence tags (ESTs) clusters and (5) sequence-based marker data sets, including shotgun sequences of 1BS [31], BAC-end sequences (BESs) and genetic markers used to infer contig position and orientation with respect to the model grass reference genomes. The construction of the 1BS physical map and the corresponding resources provide a solid base for BAC-by-BAC sequencing of this chromosome arm, as well as vital support for ongoing and future projects for map-based cloning of genes and quantitative trait loci (QTLs) mapped to 1BS. Moreover, the physical map of chromosome 1BS provides an insight into its unique gene space organization and evolution.

## Results

### Automated physical map assembly

**Figure 1:**
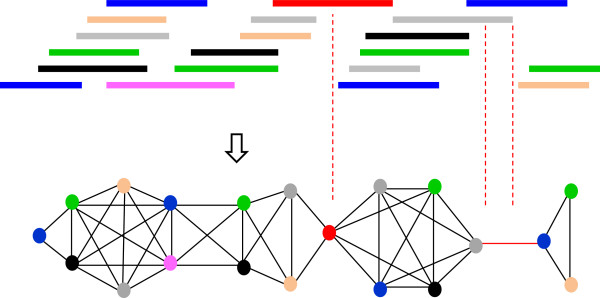
**Network representation of clone overlaps.** Top: physical clone overlaps. Bottom: network representation of clones (nodes) and clone overlaps (edges). Colors are used to clarify the correspondence between physical and network representations of clone overlaps. Weak connections (Q-clones and Q-overlaps) caused by low coverage are marked in red. Q-clone, questionable clone; Q-overlap, questionable overlap.

A wheat BAC library containing 55,296 BAC clones (average size 113 kb) was constructed from the short arm of chromosome 1B. The DNA of 1BS was isolated using flow cytometric sorting from the CS ditelosomic line [8] and fingerprinted using the HICF SNAPshot protocol [10]. In total, 49,412 high-quality fingerprints (89% of the obtained clones) representing 14.4 1BS equivalents (= 113 kb × 49,412 × 0.81 (proportion of 1BS in the sorted fraction)/ 314 Mb) were used for contig assembly by LTC [16] and compared to an FPC assembly [12,32]. The LTC network representation of significant clone overlaps (Figure 1) was used to identify Q-clones and Q-overlaps, as well as non-linear structures contradicting the one-dimensional chromosome structure.

The assembly obtained by LTC contained fewer, longer and more reliable BAC contigs than the assembly obtained by FPC, as evidenced by the following comparisons. The LTC version of the 1BS physical map contained 1,075 contigs, of which 254 contigs had ≥6 clones, compared with 1,793 contigs obtained by FPC, 518 of which had ≥6 clones (Additional file 1). The N50 value was more than twice as high for the LTC assembly than for the FPC assembly (2,430 versus 1,033 kb) and L50 was less than half (35 versus 81 for contigs with ≥6 clones) (Table 1). The average size for contigs with ≥6 clones was twice as high for LTC than for FPC (1,076 versus 531 kb). The maximum contig size was approximately 7.0 Mb in the LTC map compared to only approximately 4.7 Mb in the FPC map (Table 1). The estimated coverage of 1BS was slightly higher for FPC (88%) than for LTC (87%). This difference can be explained by the fact that the FPC contigs included approximately 1,500 additional clones considered to be Q-clones and therefore excluded from the LTC assembly. The current FPC and LTC assemblies are available from the Unité de Recherche Génomique Info (URGI) genome browser for the wheat 1BS physical map [33]. In total, 2,283 and 3,827 clones were selected as the FPC and LTC versions of the minimal tiling path (MTP), respectively. Re-arraying into three-dimensional pools and BAC-end-sequencing were conducted for 6,447 1BS clones selected using LTC and included MTP clones, clones from short contigs and Q-clones used to cure gaps in the resulting physical map (see section Physical scaffolding of 1BS BAC contigs).

**Table 1:** Comparison of FPC with LTC assemblies of 1BS BAC contigs and scaffolds

**Contigs with **≥**6 clones**	**FPC contigs**	**LTC contigs**	**LTC scaffolds**
Average clone number in contigs	65.8	133.3	565.5
Maximal clone number	580	868	2,587
Average contig size (kb)^a^	531	1,076	4,564
Maximal contig size (kb)^a^	4,681	7,006	20,881
N50 (kb)^a^	1,033	2,430	8,515
L50	81	35	11
N90 (kb)^a^	250	670	2,260
L90	292	115	34
Coverage in length (Mb, % of 1BS)^a^	275 (88%)	273 (87%)	260 (83%)

^a^Calculated based on the number of clones, mean clone length and coverage.

### Physical scaffolding of 1BS BAC contigs

**Figure 2:**
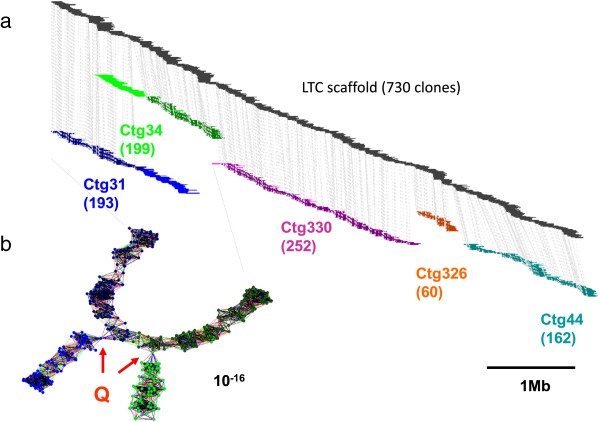
**LTC scaffolds produced by end-to-end merging and comparison with FPC contig assembly. (a)** The LTC scaffold with 730 clones resulting from a merge of five contigs is shown at the top of the figure as a linear representation. The five FPC contigs that cover the corresponding region are displayed underneath. The gray lines connect to the start points of the corresponding BACs. The blue (Ctg31) and the green (Ctg34) FPC contigs are two examples of false merging of unrelated contigs by Q-clones that were resolved in the LTC scaffold assembly. **(b)** Network representation of the assembly of contigs Ctg31 (blue) and Ctg34 (green) by LTC. Vertices correspond to clones and edges correspond to significant clone overlaps. It can be seen that the parts of the contigs excluded from the LTC scaffold are connected by only a single Q-clone, while LTC’s assembly is validated by multiple connections that exist between other clones of these two contigs. BAC, bacterial artificial chromosome; FPC, FingerPrinted Contigs; LTC, Linear Topology Contig; Mb, megabase; Q-clone, questionable clone.

**Figure 3:**
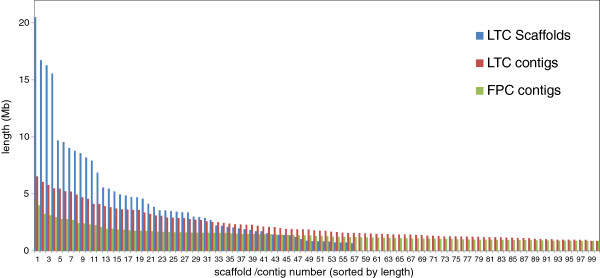
**Comparison of contig lengths of FPC and LTC assemblies and scaffolding.** FPC contigs are marked in green, LTC contigs in red and LTC scaffolds in blue. Only the longest 100 contigs are shown for each assembly. LTC-based contigs are longer than FPC contigs. The 57 constructed LTC-based scaffolds covered approximately 83% of the entire 1BS. FPC, FingerPrinted Contigs; LTC, Linear Topology Contig; Mb, megabase.

The initial LTC assembly was performed with a Sulston score cut-off of 10^-15^ to 10^-33^, excluding Q-clones and Q-overlaps. In total, 36,635 clones (74% of 49,412) were assembled into 1,075 reliable contigs with ≥2 clones (254 of which had ≥6 clones). Manual contig elongation and end-to-end merging using LTC produced a chain of contigs referred to as scaffolds hereafter (Figure 2). This procedure was conducted with a less stringent cut-off (10^-15^) and also used Q-clones and Q-overlaps for curing gaps in the assembly. In total, 1,057 BAC contigs were merged into 57 scaffolds consisting of 32,231 clones. This number represents 87% of the clones included in the initial LTC contigs and approximately 300 additional clones that were subsequently used for the scaffold assembly. Scaffold sizes varied from 1.2 to 20.8 Mb with an average of 4.6 Mb (N50 = 8.5 Mb; L50 = 11 scaffolds, Table 1 and Figure 3). These scaffolds cover approximately 260 Mb, which corresponds to 83% of the 1BS arm.

The physical contig and scaffold assemblies were partially validated using PCR-based molecular markers. The presence of a PCR marker in adjacent MTP clones provides confirmation for the physical overlap of the clones. In MTP clones selected by LTC, adjacent clones are noticeably overlapped. The Sulston score was <10^-15^, indicating that >30% of the bands are shared, that is, on average, >30% overlap of the physical length. In fact, we found an average of approximately 2.4 positive MTP clones for each marker, indicating that the level of the actual MTP clone overlaps is even higher than expected by the theoretical predictions.

### Establishment of marker resources and assignment of markers to contigs and scaffolds

Integration of gene-derived markers into 1BS physical contigs and scaffolds was achieved by hybridizing the 57 three-dimensional 1BS MTP BAC DNA pools (6,447 clones) to the NimbleGen 40 K array containing 39,179 wheat NCBI UniGenes developed by Rustenholz *et al*. [34]. After signal quantification, normalization and data deconvolution, 1,063 UniGenes were unambiguously assigned to 1,568 1BS clones (Additional file 2) from the scaffolds representing 262 Mb of 1BS. The clone position of a random sample of 82 markers obtained based on this hybridization strategy was confirmed by PCR.

One of the strategies used for anchoring BAC contigs was to employ PCR markers developed from the 1BS sequence data, as well as publicly available markers. We have developed a large number of primer pairs that can be converted into PCR markers for 1BS mapping using 454 sequence data of sorted 1BS chromosomes [35] and the 1BS BES generated in the current study. The 1BS gene-related sequences, identified based on the group 1S GenomeZipper [35], were used to construct 361 gene-related primer pairs (Additional file 3). In addition 40 primer pairs for gene-related markers (Additional file 3) were designed based on homology with barley UniGenes [36]. Insertion-site based polymorphism (ISBP) and simple sequence repeat (SSR) markers were developed by analyzing 727 Mb of 1BS 454 sequence and 9 Mb of the BES of the MTP clones with the IsbpFinder and SSRDesign Perl scripts [37]. Primer pairs for 44,729 high-quality ISBP markers and 10,543 SSR markers were constructed and are now available for mapping for chromosome 1BS (Additional file 3). Using BES, primer pairs for 4,035 ISBPs and 819 SSRs were developed (Additional file 3). The advantage of using BES-based markers is that their position in the corresponding 1BS contig is already known. As a proof of concept, we assigned 102 newly developed and publicly available PCR markers (15 SSRs, 16 ISBPs and 71 EST based) to 32 BAC contigs corresponding to approximately 18 Mb of 1BS (Additional file 2). The anchoring of 32 contigs with these markers was verified by PCR amplification of 65 individual MTP clones. These 32 contigs were incorporated into 23 scaffolds covering 161.9 Mb of 1BS. Thus, the scaffolding strategy made it possible to anchor 52% (161.9 of 314 Mb) of the 1BS DNA using only 102 PCR markers.

Finally, *in silico* anchoring of the sequences to particular BACs, based on unambiguous sequence similarities, enabled us to assign markers to contigs and scaffolds covering 237 Mb (75% of 314 Mb) of the 1BS chromosome. BAC-end sequencing of 6,447 1BS MTP clones yielded 12,894 sequences, with a total size of 9 Mb, which is 3% of 1BS. The BESs provide, on average, one sequence tag for every 25 kb. Non-repeat masked BESs were aligned with 1,776 1BS Illumina survey sequence contigs [31] obtained due to their similarity (e-value < 10^-25^) with UniGene sequences that gave signals from the NimbleGen hybridization (Additional file 4). In total, 1,641 Illumina contigs (11.96 Mb) were integrated into the 1BS physical map based on very high similarity (>600 bp and at least 99% sequence identity) with non-repeat masked BESs of the MTP clones and homology with the group 1S GenomeZipper (Additional files 2 and 5). A comparison of the 1BS BESs (after repeat masking) to group 1S GenomeZipper revealed homology with 244 gene sequences (e-value < 10^-10^; 1.9% of BESs), which made it possible to integrate these clones into a 1BS physical map based on the 1S zipper position.

### Chromosome 1BS deletion bin mapping

To identify chromosomal intervals determined by 1BS deletion bins [38], we ordered wheat ESTs with known deletion bin position [39] using the physical coordinates of the closest homologs on *Brachypodium* chromosome 2 (Bd2), rice chromosome 5 (Os5) and sorghum chromosome 9 (Sb9), which make up the group 1S GenomeZipper (Additional file 5). This order was used to define five synteny blocks for the model grass genomes corresponding to 1BS deletion bins. We have designated these blocks as established bins I to V (Additional file 6).

**Figure 4:**
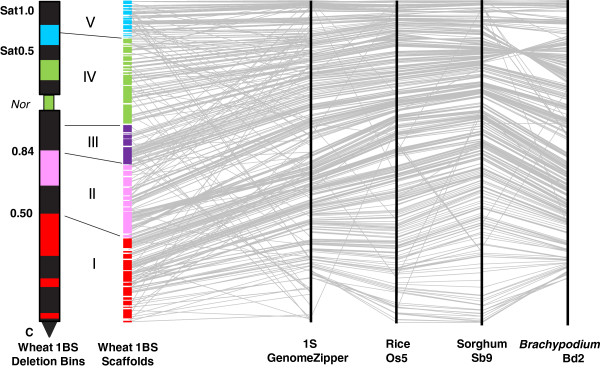
**Integrated physical map of the short arm of wheat chromosome 1BS.** The anchored 1BS scaffolds are shown as colored rectangles, with the rectangle length corresponding to the estimated scaffold length scaled to the total length of 1BS. Each color corresponds to one of the five bins (I to V) established based on the synteny of 1BS and *Brachypodium* Bd2, rice Os5 and sorghum Sb9 orthologous regions. The black segments correspond to the heterochromatic regions identified by C-bending. The fraction lengths from the centromere or the *Nor* are indicated on the left of the chromosome arm. Four black vertical lines (on the right) correspond to 1S GenomeZipper (from 0 at the bottom to 10 Mb at the top, scaled according to *Brachypodium* Bd2), rice Os5 (from 0 at the top to 8.8 Mb at the bottom), sorghum Sb9 (from 0 at the top to 15.5 Mb at the bottom) and *Brachypodium* Bd2 (from 30 Mb at the bottom to 40 Mb at the top). Each gray line starting in a wheat scaffold and ending on the 1S GenomeZipper represents a gene assigned to the scaffold for which an orthologous gene on 1S GenomeZipper was identified. The homologous relationships between Os5, Sb9 and Bd2 are also shown by gray lines. Mb, megabase; *Nor*, nucleolar organizing region.

The borders of bins I and II were defined based on EST mapping and synteny along deletion bins C-1BS10-0.50 and 1BS10-0.50-0.84, respectively. There was poor EST mapping and a large inversion in *Brachypodium* Bd2 in the interval corresponding to the nucleolar organizing region (*Nor*) and the proximal part of the 1BS satellite, covered by cytogenetic deletion bins 1BS.sat-0.31 and 1BS.sat19-0.31-0.50 and partially by 1BS9-0.84-1.06 and 1BS.sat18-0.50-1.00. Hence, we combined information on EST assignment to 1BS bins and ESTs mapped to scaffolds to identify synteny blocks for the model grass genomes corresponding to that interval. Established bin III corresponds to the proximal part of the deletion bin 1BS9-0.84-1.06. Bin IV covers the distal part of bin 1BS9-0.84-1.06 as well as 1BS.sat-0.31 and 1BS.sat19-0.31-0.50 and partially covers the proximal part of bin 1BS.sat18-0.50-1.00. For convenience, when providing the statistics for bins, we referred to formal bin IV using the name of a narrower deletion bin 1BS.sat-0.50 and when referring to the estimated bin position for markers, we used the name of a wider deletion bin 1BS9-0.84-sat1.00. Bin V covers the distal part of deletion bin 1BS.sat18-0.50-1.0 (Figure 4, Additional files 5 and 6). This approach enabled us to assign 457 1BS Illumina sequence contigs (28% out of 1,641) [31] and 383 (36% of 1,063) UniGenes, mapped to 1BS MTP clones by hybridization, to the established 1BS deletion bins based on their positions in group 1S GenomeZipper (homology with e-value < 10^-10^) (Additional files 2 and 5).

Furthermore, 121 PCR markers (68 ISBPs from the BES of MTP clones and 53 ESTs found due to colinearity between wheat and model grasses) developed in the current study were also used for deletion bin mapping. Of these, 29 (24%) markers were specific to 1BS when tested on nulli-1B-tetrasomic-1A, ditelosomic-1BS and 1BL lines, and the DNA of sorted 1BS and 1AS chromosomes. These markers, together with 15 1BS-specific SSRs from the GrainGenes database [40], were assigned to one of nine 1BS deletion bins: C-1BS-0.35, 1BS1-0.35-0.50, 1BS10-0.50-0.54, 1BS20-0.54-0.84, 1BS9-0.84-1.06, 1BS.sat-0.31, 1BS.sat19-0.31-0.50, 1BS.sat18-0.50-0.52 and 1BS.sat4-0.52-1.00 [38] (Additional file 2).

### Anchoring, ordering and orientation of 1BS physical contigs and scaffolds

The ordering and orientation of BAC contigs and scaffolds along the 1BS arm was carried out based on the assumption that wheat and sequenced model grass genomes share extensive colinear relations. The constructed large 1BS segments (for example, the 1.2 to 20.8 Mb scaffolds, average length of 4.6 Mb) enabled us to test and validate this assumption. The closest homologous gene sequences shared between *Brachypodium* Bd2, rice Os5 and sorghum Sb9 and orthologous to 1BS were ordered using the 1S GenomeZipper [25,26,35]. The observed similarity of gene orders in orthologous intervals was extrapolated to define the order of 457 1BS gene-containing Illumina sequence contigs [31] and 383 UniGenes mapped to 1BS by the NimbleGen microarray hybridization approach. The 1BS BAC contigs to which gene-derived markers were assigned were ordered according to the positions of the closest homologs in the 1S GenomeZipper (Figure 4, Additional files 2 and 5). Using this approach, we were able to anchor 53 of the 57 (93%) 1BS scaffolds to the 1S GenomeZipper and to the 1BS deletion bin map. Five contigs and two scaffolds (3% of clones) were anchored to the 1BS deletion bin map only, using ISBP and SSR markers. The anchored physical map of 1BS includes 189 of 254 contigs (74%) with ≥6 clones, covering 241.1 Mb (77.4%) of the chromosome (Figure 4 and Table 2). This map contains 2,438 markers, 903 of which were mapped physically and 1,535 were mapped *in silico*, with an average density of 10 markers per Mb. The position on group 1S GenomeZipper was identified for 620 (24%) of these markers. The final 1BS physical map is available at the wheat genome browser of the URGI website [33].

**Table 2:** Scaffold and marker distribution along the short arm of wheat chromosome 1B

**Wheat 1BS deletion bins**	**Established wheat 1BS deletion bins**	**Assigned scaffolds (Mb)**	**Number of markers**	**Marker density (marker/Mb)**
1BS.sat18-0.50-1.0^a^	V	26.8	340	12.7
1BS.sat5-1.06-0.50	IV	62.2	585	9.4
1BS9-0.84-1.06^a^	III	47.4	423	8.9
1BS10-0.50-0.84	II	35.3	369	10.5
C-1BS10-0.50	I	69.4	724	10.4
Total in scaffolds		241.1	2,438	10.1
In BAC clones^b^			501	
Total in scaffolds and in BAC clones			2,939	

^a^Deletion bins partly covered by bin IV as shown in Figure 1; ^b^BAC clones that were not included in scaffolds.

The development of the anchored 1BS physical map enabled us to compare the gene order in 1BS and in orthologous intervals of the model grass genomes. The global and local gene orders established for 1BS were found to be similar to those of corresponding homologous genes in Bd2, Os5 and Sb9. In total, 73.3% of the gene-related markers mapped to 1BS were in the same order as the homologous genes in the orthologous regions of at least two of the three utilized model grass genomes (Figure 4, Additional file 5). Nevertheless, some differences in the gene order between 1BS and the orthologous intervals of Bd2, Os5 and Sb9, detected using 1BS BAC contigs and scaffolds, indicate the presence of breaks in colinearity. Accordingly, our results show that a large distal inversion occurred between *Bradi2g37120* and *Bradi2g38650* on Bd2, relative to the 1BS interval covered by Scaff37 and part of Scaff13 (Figure 4, Additional file 5). This inversion corresponds to the region of a large inversion in *Brachypodium* Bd2 relative to rice Os5 (between *LOC_Os05g02140* and *LOC_Os05g04370*) and sorghum Sb9 (between *Sb09g001370* and *Sb09g002790*). Based on the homology of at least two of the three model grass genomes, we identified six additional colinearity breaks between wheat 1BS and Bd2, Os5 and Sb9 involving a total of 106 gene-derived markers (Figure 4, Additional file 7). Furthermore, 60 additional gene-derived markers were found to be located in non-colinear positions in orthologous regions of only one of the three genomes. The non-colinear gene-markers were located in the following six scaffolds, listed from centromere to telomere according to the parallel position on Bd2: Scaff12 (from *Bradi2g31967* to *Bradi2g32230*), Scaff4 (*Bradi2g32250* to *Bradi2g33370*), Scaff24 (*Bradi2g34560* to *Bradi2g35467*), Scaff55 (*Bradi2g37650* to *Bradi2g37750*), Scaff48 (*Bradi2g39700* to *Bradi2g39860*) and Scaff27 (*Bradi2g40030* to *Bradi2g40120*). The synteny profiles of *Brachypodium* Bd2, rice Os5 and sorghum Sb9 are quite similar in these chromosomal regions.

### Effect of restriction site distribution along 1BS on BAC contig length

**Figure 5:**
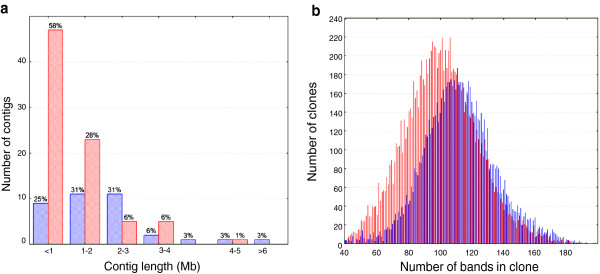
**Gradients of contig length and clone band number along the 1BS chromosome. (a)** Distribution of length of contigs mapped to the centromeric part of 1BS (deletion bin C-1BS10-0.50, shown in blue) compared to contigs mapped to the telomeric region (deletion bins 1BS.sat5-1.06-0.50 and 1BS.sat18-0.50-1.0, shown in red). **(b)** Distribution of band number per clone in centromeric (blue) and telomeric (red) regions. BAC, bacterial artificial chromosome; Mb, megabase.

We observed that, on average, contigs in centromeric regions (mapped to bin C-1BS10-0.50) contain twice as many BAC clones (229.5) as those located in the telomeric region (101.2, mapped to bins 1BS.sat-0.50 and 1BS.sat18-0.50-1.00; Figure 5a). This can be partially explained by the fact that the telomeric region contains more gene-related sequences than the centromeric one; hence, short contigs from the telomeric region have a higher chance of being mapped than similarly sized contigs from the centromeric region. The lower coverage of the physical map in the centromeric region compared to the telomeric region (Figure 4 and Table 2) provides some support for this hypothesis.

Another possible hypothesis is that HICF fingerprinting has a lower chance of detecting clone overlaps in the telomeric region than in the centromeric region. The average number of bands per clone was found to be 112.1 in the centromeric region compared to only 96.7 in the telomeric region (Figure 5b). This difference could result from a variation in physical clone length caused by the uneven distribution of *Hind*III restriction sites, used for library construction, along the chromosome. Based on the Illumina shotgun sequence contigs anchored to 1BS physical scaffolds, we found that indeed *Hind*III restriction sites are more commonly present in the centromeric region (50.1 sites per 100 kb) than in the telomeric region (42.8 per 100 kb). Similar differences in the frequency of *Hind*III restriction sites between telomeric and centromeric regions were observed for all chromosomes of *Brachypodium*, rice, sorghum and maize (data not shown). For the enzymes *Hae*III, *EcoR*I, *BamH*I, *Xba*I, and *Xho*I used for HICF fingerprinting, the distribution of restriction sites in the sequenced genomes was close to uniform (not shown). In contrast, 1BS sequences had a lower frequency of *Hae*III, *Bam*HI and *Xho*I restriction sites in the telomeric than in the centromeric region (349.3, 20.4, and 18.9 per 100 kb versus 422.1, 27.2 and 25.5 per 100 kb, respectively). Hence, clones in the telomeric region are putatively longer but have fewer HICF bands because of the lower frequency of enzyme restriction sites used for fingerprinting. This means there is a lower chance of detecting clone overlaps in the telomeric region than in the centromeric region, resulting in the contigs derived from the 1BS centromeric region being twice as long.

Therefore, we conclude that both of our hypotheses about the possible reasons for the observed gradient of contig length along 1BS – that is, the higher probability of mapping short contigs in the telomeric part and the higher information content of HICF fingerprinting in the centromeric region – fit the data.

### Scaling of wheat 1BS arm based on physical map

Chromosome 1BS BAC contigs and scaffolds anchored to 1S GenomeZipper and deletion bins made it possible to scale 1BS relative to *Brachypodium* Bd2 and to estimate 1BS deletion bin sizes. Considering the coverage of 1BS by anchored contigs (77.4%), the physical size, *L*_W_, of the wheat 1BS arm corresponding to a segment on Bd2 with length *L*_B_ can be estimated as:

$$L_{W}∼1/0.774\times L_{1\mathrm{BS}}/L_{1S\left(\mathrm{Bd}2\right)}\times L_{B}$$

where *L*_1BS_ = 314 Mb is the length of 1BS and *L*_1S(Bd2)_ ~ 10 Mb is the length of the Bd2 segment corresponding to the entire 1BS arm.

Based on cytological estimates [38], C-1BS10-0.50, 1BS10-0.50-0.84, 1BS9-0.84-1.06, 1BS.sat-0.31, 1BS.sat19-0.31-0.50 and 1BS.sat18-0.50-1.00 are approximately 33%, 22%, 11%, 10%, 7% and 17% of 1BS, respectively. Due to the uncertain identification of the corresponding intervals on group 1S GenomeZipper for deletion bins 1BS.sat-0.31 and 1BS.sat19-0.31-0.50, we combined all the data obtained for these bins and parts of bins 1BS9-0.84-1.06 and 1BS.sat18-0.50-1.00 to the synteny block designated as bin IV comprising approximately 27% of the length of 1BS (Figure 4).

Our estimates of bin sizes, based on the scaffolds anchored to these bins, are consistent with the proportions of their length estimated cytologically (Table 2). A comparison of 1BS size with Bd2 revealed that there had been an average of 30-fold expansion in the wheat chromosome relative to *Brachypodium*.

We estimated the relative physical distances between the contigs by their position within deletion bins along 1BS and compared them with the relative distances of the orthologous region on *Brachypodium* Bd2. This comparison revealed a non-uniform distribution of expansion along 1BS from the centromere to the telomere: the increase in size was twice as high in the telomeric part (approximately 45×) than in the centromeric part (approximately 20×) (Figure 4). This finding contrasts with the uniform expansion in chromosomes 3B of wheat [41] and 3DS of *Aegilops tauschii*[42] compared to rice. Nevertheless, it is consistent with the non-uniform expansion seen when comparing rice and sorghum, where the increase in the size of the sorghum genome was entirely accounted for by the increase in the proximal heterochromatic regions [15].

### Gene space organization along chromosome 1BS: estimation of 1BS gene content

Hybridization of a NimbleGen 40 K microarray with three-dimensional pooled MTP clones of 1BS revealed 3,878 UniGenes that were putatively mapped to 1BS. For these UniGenes, at least two positive MTP clone pools per UniGene, corresponding to coordinates of specific clone(s) address on the physical contig, were identified and confirmed by the maximum likelihood method (see Materials and methods). The probability of obtaining ≥2 positive pools for a UniGene by chance alone is very low (0.006). Moreover, the availability of two known coordinates simplifies the process of obtaining the physical position of these UniGenes on 1BS by PCR screening of the three-dimensional pools, especially for UniGenes from the intersection of two or more MTP clones.

Wicker *et al*. [35] estimated that the total number of 1BS genes is 3,664 based on alignment of the 727 Mb of 454 sequence data of 1BS flow-sorted chromosomes against 25,468 *Brachypodium* genes that have DNA homology support from rice and sorghum. The exact proportion of 1BS genes on the NimbleGen 40 K chip is generally unknown. Nevertheless, the similarity of the estimates for 1BS gene content obtained by analyzing the whole-chromosome 454 sequence data and the hybridization results suggest that 1BS has approximately 3,600 to 3,800 genes. The correspondence of these independent estimates is indeed very good, especially considering that it is impossible to distinguish between genes and pseudogenes (gene fragments and non-functional gene copies) in the short-read data sets or by the microarray hybridization used in the current study. Full sequencing and ordering of non-repetitive chromosomal fraction is required to resolve gene/pseudogene presence and evolutionary dynamics.

### Two-fold increase in gene content towards 1BS telomere

**Figure 6:**
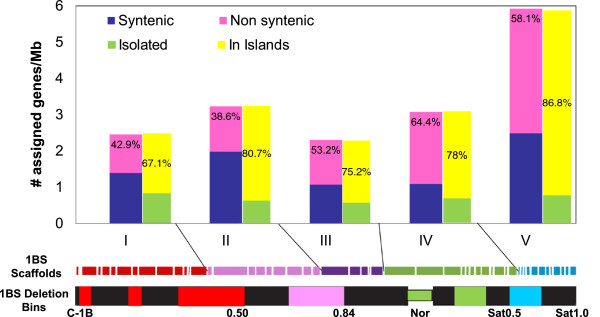
**Gene density distribution along the short arm of wheat chromosome 1B.** The anchored 1BS scaffolds are shown as colored rectangles. The scaffold lengths are scaled to the total length of 1BS. Each color corresponds to one of the five bins (I to V) established based on the synteny of 1BS and *Brachypodium* Bd2, rice Os5 and sorghum Sb9 regions. The black segments correspond to the heterochromatic regions identified by C-bending. The fractional length from the centromere or the *Nor* is indicated below the chromosome arm. The proportion of syntenic genes (blue bars) and non-syntenic genes (pink bars and percentage within) is shown for each deletion bin. The density of isolated genes (green bars) and genes organized in islands (yellow bars and percentages within) are shown for each deletion bin. Mb, megabase; *Nor*, nucleolar organizing region.

We investigated 1BS gene space organization by estimating the distribution of mapped UniGenes along 1BS. For this analysis, we considered the 721 UniGenes that have only one possible clone address on one of the 160-long 1BS contigs (covering 239.3 Mb or 76.2% of 1BS), thereby avoiding any potential inaccuracy caused by Q-clones. Mapping of these 721 loci to 1BS contigs resulted in their assignment to five established 1BS deletion bins defined by corresponding segments on 1S GenomeZipper (Table 2, Additional files 2 and 5). Examination of gene space organization along the five deletion bins of chromosome 1BS revealed that the gene density per unit length of the physical map increased from an average of 2.5 genes/Mb in the centromeric region to 5.9 genes/Mb in the telomeric region (Figure 6).

Mapping of the UniGenes to 1BS contigs enabled us to evaluate the level of synteny between 1BS and orthologous regions of *Brachypodium* Bd2, rice Os5 and sorghum Sb9. Out of 721 genes mapped to 1BS scaffolds, 329 (46%), 266 (37%) and 256 (36%) were found to be syntenic on Bd2, Os5 and Sb9, respectively (Additional file 5). The percentage of synteny of 1BS with Bd2 was higher than with Os5 and Sb9, which confirms that *Brachypodium* is closer to wheat than rice or sorghum [13,43]. The proportion of 1BS syntenic genes is much lower than that reported for 3B (58% and 65% for rice and *Brachypodium*, respectively [34]). Furthermore, the proportion of non-syntenic genes, based on comparisons of 1BS with Bd2, Os5 and Sb9, increased from 42% in the centromeric bin C-1BS10-0.50 to 58% in the telomeric bin 1BS.sat18-0.50-1.00 covering the proximal part of 1BS satellite (Figure 6).

To further explore the pattern of gene distribution along the 1BS chromosome, we tested for gene clustering. Assuming a random gene distribution, the 721 UniGenes should be uniformly distributed along large scaffolds (with length >1 Mb) covering 253 Mb. To test whether this assumption fits our data, we subdivided these long contigs into 1,000 non-overlapping intervals and determined the number of UniGenes in each interval. If the gene distribution were random, the expected number of intervals (each of which is 0.253 Mb in length, corresponding to more than two clone lengths) with zero, one and two or more UniGenes, would be 1,000 · (1 – 0.001)^721^ = 486.1, 1,000 · 721 · 0.001 (1 – 0.001)^720^ = 350.4 and 1,000 – 486.1 – 350.4 = 163.5, respectively.

**Figure 7:**
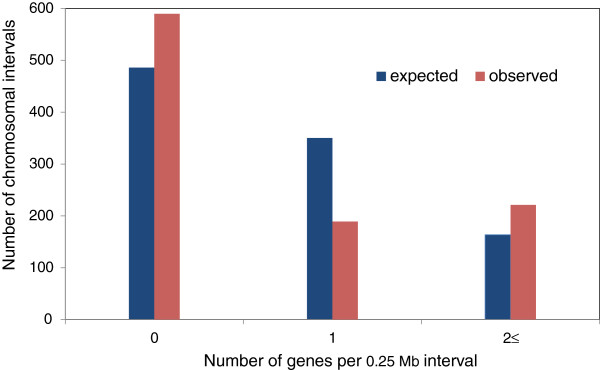
**Non-random distribution of gene density in 1BS.** The observed distribution of 0.253 Mb 1BS intervals containing no, single or at least a pair of genes was compared to the expected random distribution; an excess of intervals with two or more genes was revealed. kb, kilobase; Mb, megabase.

We consider two genes to belong to a gene island if the physical distance between them is not more than a single-clone length. Hence, if two UniGenes are found in the same clone or in a pair of significantly overlapping clones, they will be considered as members of the same gene island. In total, we found 189 of 0.253 Mb intervals with only one UniGene and 221.1 with two or more UniGenes, that is, gene islands, covering approximately 55.9 Mb. The total length of empty segments amounts to 589.9 intervals (= 1,000 – 189 – 221.1) of 0.253 Mb. By comparing the observed distribution of intervals with zero, one and ≥2 UniGenes (589.9, 189 and 221.1) with the expected distribution under the assumption of random distribution (486.1, 350.4 and 163.5) we concluded that gene distribution along the 1BS chromosome is not random (χ^2^ = 178.5, degrees of freedom = 2, *P* < 10^-10^) and there is an excess of gene-rich regions (with 2 to 20 genes, Figure 7). The clustering of genes in many small islands as found here for chromosome 1BS is in general agreement with the gene organization found in chromosome 3B [34,41,44], as well as for *Aegilops tauschii*, the diploid progenitor of the wheat D genome [45,46].

## Discussion

The huge size (17 Gb) of the bread wheat genome, its polyploid nature and the extremely high proportion (>80%) of repetitive elements, present a challenge to the construction of a reliable reference genome sequence for wheat. Therefore, the IWGSC has approached sequencing through the construction of BAC-based physical maps of individual chromosome arms. The significance of physical map integration in whole genome sequencing of large cereal genomes was recently demonstrated for barley [27]. Following the IWGSC chromosome-by-chromosome approach, we have constructed the first complete physical map of chromosome arm 1BS and studied its gene space organization and evolution.

### Significant increase in BAC scaffold length using the LTC adaptive clustering strategy

We constructed a physical map of the 1BS arm using fingerprinting and contig assembly of 49,412 BAC clones (17× of 1BS). We used LTC [16] for contig assembly and scaffolding and compared LTC and FPC assemblies [12,32]. The LTC adaptive clustering strategy developed in our group dramatically improved assembly of the 1BS physical map and resulted in half as many contigs (which were, therefore, twice as long) compared to those constructed by FPC. Moreover, the LTC program enabled us to elongate 254 BAC contigs and to merge them into only 57 very long scaffolds (average size 4.6 Mb, maximal size 20.8 Mb) with an N50 value that was more than eightfold higher than the one obtained from FPC assembly (8,515 kb versus 1,033 kb). A comparison of wheat chromosome physical maps published to date (for example, 1AS [47], 1AL [48], 1BS (this study) and 1BL [49]) clearly shows that LTC assemblies of BAC contigs are approximately twice as long as those obtained by FPC. Furthermore, while the assembly of 3B contains 1,036 contigs (approximately 800 Mb, 80% of 3B [22]), the 1BS assembly contains only 57 scaffolds, which represent more than 83% (260 Mb) of 1BS. Similar differences can be observed when comparing the final assembly of 1BS with that of 1AS [47], 1AL [48] and 1BL [49].

### Comparative genomics as a strategy for assembly, validation and contig anchoring

The high coverage (77.4% of 1BS) of the physical map, assembled and anchored in the current study, was achieved using several complementary strategies: (1) merging of contigs into scaffolds using LTC, (2) marker assignment to contigs and anchoring to 1BS deletion bins and (3) comparative genomics based on synteny between wheat and model grass genomes. This approach resulted in the anchoring of 53 1BS LTC scaffolds, covering 243.1 Mb of 1BS, to deletion bins and to 1S GenomeZipper. The 1BS physical map was further enhanced by integrating the information from 1,641 Illumina survey sequence contigs [31] and 12,894 BESs. In total, 2,438 markers were placed on the anchored physical map, most of which (87.5%) are gene-related markers integrated by direct molecular (PCR and microarray) or *in silico* analyses (Table 2). In addition to providing valuable genetic information, these markers were extremely useful for validating the LTC assembly based on synteny with fully sequenced model grass genomes: *Brachypodium*, rice and sorghum using the GenomeZipper approach [25,26]. We found that 73.3% of the gene-related markers mapped to 1BS BAC clones and arranged in scaffolds were in the same order as the homologous genes in the orthologous regions of model grass genomes. The consistency in gene order observed between the 1BS physical map and the orthologous regions in model grass genomes verifies the reliability of the LTC scaffold assembly based on end-to-end merging of contigs.

The synteny-based integration of wheat BAC contigs using sequence information of model grass genomes was especially helpful for regions with limited resolution of wheat genetic maps due to suppressed recombination in the centromeric and subcentromeric regions of 1BS. The genetic maps of the short arms of B genome chromosomes of wheat are derived almost exclusively from recombination in the distal 25% to 30% of their physical length [18]. Furthermore, SSR mapping showed that 60% of 1BS is represented by only approximately 2% of the genetic map [50]. In the current study, a total of 1,366 gene-related markers were located in regions with low recombination frequency, thus representing genes with limited accessibility based on genetic mapping. Anchoring scaffolds to 1BS deletion bins and 1S GenomeZipper enabled us to overcome the limitations imposed by meiotic mapping. Moreover, the anchored physical map enabled us to identify the exact physical position of 1,074 gene-related markers in low-recombination regions, and 553 genes in high-recombination regions (62% of mapped gene-related markers in total) for which no homologous sequences in the orthologous regions of model grass genomes were identified. Map-based cloning studies of important wheat genes (for example, *Gpc-B1*[51,52] and *Yr36*[53]) have shown that orthologs for the targeted wheat genes were lacking in rice within otherwise well-preserved colinear genome segments. Several classes of genes that are either unique to wheat (for example, end-use quality) or rapidly evolve (such as disease resistance genes (*R*-genes)) [54], will likely be among those that have no matched homologs in the model grass genomes.

It is believed that gene distribution along chromosome arms with no regions that are devoid of genes is common for wheat chromosomes, as was shown by microarray hybridization-based mapping and sequence homology studies of chromosomes 3B [22] and 1BS (current study). Our 1BS physical map provides a rich source of gene-related markers for map-based cloning of genes, even in low-recombination regions. Nevertheless, complementary strategies, such as radiation hybrid mapping or genome-wide association analysis may be required, in addition to the 1BS physical map, for gene identification and gene isolation studies.

### Gene space organization of the 1BS chromosome

The current physical map served as a basis for transcriptional mapping of 1BS, which was achieved by hybridizing the NimbleGen 40 K wheat UniGene microarray with 1BS MTP BAC pools. The transcriptional map contained 721 loci, which were used to study the gene space organization along 1BS and its evolution. The transcriptional map confirmed that the gene space spans the entire length of 1BS, and displays a gradient of gene density with a two-fold increase from the centromere to the telomere. Furthermore, 76% of the genes were organized in small gene islands composed of two or three genes. A similar pattern of increase in gene density from proximal to distal regions of 1BS arm was inferred by Peng *et al*. [39], who mapped ESTs to 1BS deletion bins. Choulet *et al*. [41] and Rustenholz *et al*. [34] reported a similar gradient of gene density along wheat chromosome 3B. Gene density in many grass genomes increases overall from the proximal towards the distal regions of chromosome arms. This gradient is heterogeneous and regions of higher and lower gene density are superimposed on it. In fact, such gradients have been observed in all of the grass genomes sequenced to date [45].

Higher gene density estimates were obtained by restriction fragment length polymorphism (RFLP) hybridization with ESTs than by microarray hybridization of BAC clones for the telomeric bins of chromosome 3B [44]. This difference was explained by the inability of microarray hybridization to detect tandemly duplicated genes. Similarly, gene density in 1BS telomeric bins was also higher when estimated by RFLP hybridization with ESTs [39] than for the microarray results obtained in the current study (normalized gene densities: 1.6 versus 1.3 per Mb, respectively). These findings suggest that our analysis may have underestimated the gene density in the telomeric part of 1BS, and an even steeper density gradient may be expected from the centromere to the telomere of 1BS.

We further estimated gene distribution along 1BS, keeping in mind the limitation of hybridization methods for detecting tandemly duplicated genes, and the inability to distinguish between functional genes and pseudogenes. Considering the full coverage of chromosome 1BS by BAC contigs, the distribution of the 721 loci mapped in the current study by hybridization to deletion bins, and the estimated gene number in 1BS (3,664 obtained by Wicker *et al*. [35] and 3,878 in the current study), the expected gene density can be extrapolated to the entire set of 1BS genes. The resulting density range was one gene per 99 to 105 kb for the centromeric bin C-1BS10-0.50, which is higher than the estimated density in the proximal bins for chromosome 3B (one gene per 184 kb) obtained by sequencing of 18.2 Mb of BAC contigs [41]. An analysis of 11 Mb of BES data for chromosome 3AS resulted in an estimate of one gene per 123 kb [55], while a density of one gene per 162 kb was found from recently sequenced DNA of a flow-sorted chromosome 5A [56]. Therefore, gene density appears to be higher in 1BS than in other wheat chromosomes for which gene content has been estimated.

A comparative analysis between the UniGenes identified on wheat chromosome 1BS and their homologs from *Brachypodium* Bd2, rice Os5 and sorghum Sb9 revealed that there was a slightly higher level of synteny for *Brachypodium*, as expected based on its more recent divergence from wheat. The proportion of UniGenes mapped to 1BS that were non-syntenic in model grass genomes was 55% to 65% compared to only 35% to 42% of genes mapped to 3B [34], 23% to 30% to 3A [55], and 20% to 45% to 1AL, 1BL and 1DL [35]. Our results support the recent findings of Wicker *et al*. [35] that the 1BS chromosome has accumulated an extremely high proportion of non-syntenic genes compared to other group 1 wheat and barley chromosomes. Wicker *et al*. [35] found that the proportion of syntenic versus non-syntenic genes in 1BS was 1:5, compared to 1:2 for 1AS and even less for the long arms of group 1 chromosomes (1:1.2 to 1:1.6). They suggested that most of the non-syntenic genes are pseudogenes, although more than half of the non-syntenic genes are still actively transcribed. In fact, 68% of the transcript clusters obtained for the barley transcriptome were so-called low-confidence genes, due to the lack of homology with the genes of model grasses and the lack of support from gene family clustering [27]. Rustenholz *et al*. [34] reported that 89% of the UniGenes, both syntenic and non-syntenic, mapped to 3B are actively transcribed. Although *bona fide* genes and pseudogenes are undistinguishable in our analysis of 1BS, many of them may represent fast-evolving genes (for example, *R*-genes) that are species specific and therefore unique to wheat.

Despite the considerable degree of conservation in gene order and content between chromosome 1BS and the orthologous regions on *Brachypodium* Bd2, rice Os5 and sorghum Sb9, distinct genomic rearrangements were identified. The anchoring of ESTs that had previously been mapped to 1BS deletion bins [39] into two large 1BS scaffolds confirms that wheat, rice and sorghum share the same order of gene markers and that chromosome inversion in the region between *Bradi2g37120* and *Bradi2g38650* is present only in *Brachypodium* Bd2. Most of the perturbations in the gene order in wheat 1BS relative to the orthologous chromosome regions of other grasses were small inversions and transpositions of gene blocks, with some non-colinear genes interspersed among the conserved blocks. These differences in gene order between 1BS and other grass chromosomes may reflect structural, chromosomal (inversions and transpositions) and gene space (single-gene duplications and deletions) evolution. The 1BS non-colinear genes with only one homolog in the orthologous regions of the three model grass genomes were interspersed within colinear gene blocks. Perturbation in the order of these genes may also be caused by a limited mapping resolution and by the fact that our analysis considered only gene-related markers with unique positions. These non-colinear genes probably reflect duplicated genes or gene fragments that moved due to transposable element (TE) activity [35,57]. While we cannot trace evolutionary events that led to the absence of colinearity for that fraction of gene-related markers, our results illustrate that true evolutionary relations can be revealed only if more than two species are compared [43,58].

The anchored physical map of chromosome 1BS enabled us to trace the distribution patterns of genomic components along its centromere-telomere axis. Our analysis revealed a slightly higher proportion of syntenic UniGenes in the centromeric and pericentromeric regions of 1BS, while the density of non-syntenic UniGenes was more than two-fold higher towards the subtelomeric and telomeric parts. The majority of genes on 1BS showed a tendency to cluster in small gene islands, reflecting a non-homogeneous expansion of the chromosome, which is a common feature of large and repetitive genomes, such as maize and wheat [41,59]. Furthermore, a non-uniform expansion in 1BS chromosome size was identified relative to model grass chromosomes (for example, Bd2), with an expansion in the subtelomeric part that was twice as high as that in the centromeric region of 1BS. Variation of genome size in grasses is caused mainly by the accumulation or loss of TEs. The formation of gene islands is explained by non-random insertion or preferential deletion of TEs in gene-rich regions [41,45]. Choulet *et al*. [41] proposed more active mechanisms for gene island formation, which involve gene duplication and translocation. A similar pattern of non-syntenic gene distribution, with a two-fold increase towards the telomeric parts, was found in chromosome 3B [34]. However, in contrast to 1BS, the increase in size of 3B was found to be homogeneous along the proximal and distal regions [41].

The non-uniform expansion of 1BS, together with the extremely high accumulation of non-syntenic genes by 1BS discussed above, suggests that the evolutionary forces that shaped the wheat genome may differ between different chromosomes of the same genome. Interestingly, the GC content (44.7%) of 1BS BESs shows a uniform distribution along the centromere-telomere axis, similar to the GC content reported for other wheat chromosomes [60]. Such GC values are expected for TE-rich sequences. Another characteristic that we found to be unique for 1BS is the non-uniform distribution of restriction sites of some of the enzymes used in HICF, which presumably resulted in shorter contigs in the telomeric regions compared to centromeric ones. This sequence composition of 1BS is distinct from other cereal genomes for which BAC physical maps were constructed, such as *Brachypodium*, rice, sorghum and maize.

### Wheat genome asymmetry and patterns of evolution in chromosome 1BS

Allopolyploid wheat species harbor considerable genetic diversity, with levels that differ between the three genomes (reviewed in [61]). The B genome is the largest of the three wheat subgenomes [8] with the largest number of genes, as estimated by shotgun sequencing (38,000, 36,000 and 28,000 genes for the B, D and A genomes, respectively [62]). The B genome exhibits higher polymorphism, a higher proportion of repetitive DNA and a lower synteny level than the A and D genomes of wheat [61,63,64].

The evidence presented here shows that 1BS evolution followed a unique pattern that differed even from other B-genome chromosomes (for example, 3B). These lines of evidence include: (1) a relatively large gene space, as reflected by a considerably higher number and density of gene-markers, (2) the accumulation of an extremely high proportion of non-syntenic genes, (3) a non-uniform size expansion along the centromere-telomere axis, (4) a non-uniform distribution of BAC contig length, with longer contigs in the centromeric region and (5) a non-uniform distribution of restriction sites (for example, *Hae*III used for HICF) along the centromeric-telomeric axis. Although we do not currently have a complete picture of the forces that have shaped the structure and evolution of chromosome 1BS, there is reason to believe that these patterns were influenced by selection pressure varying with recombination rates (for example, [65]). The most striking characteristic of 1BS, revealed here, is the steep gradient in the genomic features along the centromere-telomere axis, which is associated with the gradient in the recombination rate. This association is illustrated by the fact that 95% of the meiotic map of 1BS (for example, [50]) is represented by only 34% (82 Mb) of the scaffolds, anchored in the current study to the subtelomeric and telomeric regions of 1BS. The 45-fold increase in genome size near the telomere, relative to model grass genomes, compared to the only 20-fold expansion near the centromere, is probably associated with the remarkably high recombination rate at the telomeric region of 1BS. Other studies have shown that intergenic regions and non-coding parts of genes rapidly evolved through TE insertions and deletions caused by illegitimate recombination, double-strand break repair and unequal crossing-over [57].

In the current study, we found that the dramatic accumulation of non-syntenic genes (most likely pseudogenes) in 1BS, compared with the other studied wheat chromosomes, was much more pronounced in the telomeric region. Pseudogenes tend to evolve much faster than functional genes and therefore have the potential to accelerate genome evolution [66]. We propose that 1BS may play an important role as a potential reservoir of useful variation for selection and adaptation. The unique characteristics of the 1BS structure are in agreement with the evidence accumulated over the last decade in allopolyploid wheat, indicating that genomic asymmetry is more prevalent in this species than previously thought [61].

A striking example of the primary role of the B genome’s adaptation to stress is provided by the fact that the number of functional *R*-gene loci [67] and *R*-gene analogs (RGAs) [68], mapped to the B genome, is twice as high as that for the A or D genomes. 1BS is known to harbor numerous *R*-genes, including five functional stripe rust *R*-genes (*Yr10*, *Yr15*, *Yr24*, *Yr26* and *YrH52*[69]). Plant *R*-genes have been found to be organized in clusters of duplicated genes (for example, [66,70,71]). A high number of pseudogenes in clusters may not be directly involved in resistance, but may provide the raw material from which new gene variants can emerge [66]. Therefore, the large excess of non-syntenic genes and pseudogenes in 1BS compared to the other chromosomal arms analyzed so far may reflect 1BS’s ‘prospective’ role in the evolution of wheat’s tolerance to biotic and abiotic stresses.

## Conclusions

The integration of physical contigs into long LTC scaffolds led to the orientation of the contigs within the scaffolds and facilitated the anchoring of the 1BS physical map. The physical map of wheat chromosome 1BS presented here comprises only 57 long BAC scaffolds, which cover 83% of 1BS’s length. This is the most comprehensive map for chromosome 1BS, with the highest number of gene-related markers, spanning regions with both high and low recombination. This map enabled us to obtain new evidence showing that the mechanisms of evolution within the wheat genome may differ at the level of specific chromosomes. The excess of non-syntenic 1BS genes probably represents a reservoir of non-functional genes (pseudogenes), which serves as the raw material for the rapid generation of new functional genes in response to biotic and abiotic challenges. The physical map developed in the current study provides a solid basis for the production of a high-quality reference sequence for 1BS. A detailed study of the DNA sequence of the 1BS chromosome, together with other wheat chromosomes, will lead to further progress in our understanding of the evolution of wheat and the biology underlying genetic plasticity and, consequently, its improvement using modern molecular biology tools.

## Materials and methods

### Purification of 1BS arms by flow cytometric sorting

Liquid suspensions of intact mitotic chromosomes were prepared from a double-ditelosomic line (2*n* = 40 + 2 *t* 1BS + 2 *t* 1BL) of *T. aestivum* L. cv. CS according to [72]. The samples were stained with 4',6-diamidino-2-phenylindole (DAPI), and both the short (1BS) and the long (1BL) arms maintained in the line as telocentric chromosomes were purified simultaneously by flow cytometric sorting [73]. The 1BS arms were sorted in batches of 200,000 as described by Šimková *et al*. [74]. The purity of sorted fractions was checked regularly by fluorescent *in situ* hybridization (FISH) using probes for telomeric repeat and GAA repeat [28].

### Construction of BAC libraries

A 1BS-specific BAC library was constructed according to Šimková *et al*. [75]. Briefly, isolated high-molecular-weight (HMW) DNA was partially digested with *Hin*dIII. DNA fractions with size range of 100–200 kb were ligated into the *Hin*dIII-digested dephosphorylated pIndigoBAC-5 vector (Epicentre, Madison, WI, USA). The recombinant vector was used to transform *Escherichia coli* MegaX DH10B-competent cells (Invitrogen, Carlsbad, CA, USA). The 1BS-specific BAC library, coded as TaaCsp1BShA, was ordered into 384-well plates filled with 75 μl freezing medium consisting of 2 × yeast extract tryptone, 6.6% glycerol and 12.5 μg/ml chloramphenicol. The average insert size was estimated based on an analysis of 200 randomly selected BAC clones [28].

### Fingerprinting reaction and data processing

BAC DNA samples were extracted from the 1BS BAC library and fingerprinted using the HICF SNaPshot protocol [10] with some modifications [22]. Peak areas, peak heights and fragment sizes for each BAC fingerprint profile were collected by the ABI Data Collection program. High-quality fingerprints were obtained using the GeneMapper [76] and FPB software [77] to extract peak data, remove background and vector-related bands and detect cross-contamination of clones. For each clone, the obtained band sizes of all four colors were multiplied by 30, affined and united into a single list to fit the FPC input data format.

### BAC contig assembly

High-quality fingerprinted BACs were assembled into contigs using the following tools: (1) the FPC program [12,32] according to the *TriticeaeGenome* Consortium’s guidelines and (2) the LTC program [16]. Automatic FPC assembly was conducted with the following parameters: tolerance 12 (corresponding to 0.4 bp), gel length 54,000 (= (500 – 50) × 4 × 30) and maximal rate of Q-clones equal to 10%. Initial clustering was performed with a Sulston score cut-off of 10^-75^. Contigs with >10% of Q-clones were reassembled with an increasing cut-off stringency of up to 10^-84^. The resulting contigs were automatically end-to-end merged with a cut-off of 10^-45^ or higher and at least 50 common bands in ending clones. LTC assembly was conducted semi-automatically with the following parameters: gel length 60,000 (= 500 × 4 × 30), tolerance 12 and the total number of different bands for Sulston score calculation 2,250 (= (500 – 50) × 4 × 30 / (2 × 12)). A network of significant clone overlaps was constructed using a Sulston score cut-off of 10^-15^. Q-overlaps were excluded at a cut-off of 10^-15^ and Q-clones were excluded at a cut-off of 10^-25^[16]. An additional set of Q-clones was excluded at a cut-off 10^-30^ to split a large group of highly overlapping clones. Another set of Q-clones was excluded manually to split groups of overlapping clones into contigs with linear topology. Clones with >500 highly significant (<10^-50^) overlaps were also excluded. After excluding the Q-clones and Q-overlaps, LTC contigs were assembled automatically with a stringency cut-off of 10^-15^.

### Establishing minimal tiling paths

For FPC contigs, MTP clones were selected by FPC with the following parameters: Min FPC Overlap = 30, From End = 30 and Min Shared bands = 12. For comparison, LTC-based MTP clones were also selected for FPC contigs (see Results). For LTC contigs, MTP clones were obtained using the LTC program with a natural requirement of significant clone overlap for adjacent MTP clones (at a cut-off of 10^-15^ to 10^-33^ corresponding to approximately 30% to 50% of shared bands, depending on clone length). A list of MTP clones for LTC contigs was complemented by clones that can potentially be used for contig elongation and merging (see Results). The extended list of MTP clones was re-arrayed into three-dimensional pools and BAC-end sequenced.

### Construction of 1BS physical scaffolds

LTC contigs confirmed by significant parallel clone overlaps were manually assembled into scaffolds by the LTC program. Contig elongation and end-to-end merging were conducted at cut-offs of up to 10^-15^ using Q-clones and Q-overlaps excluded in the previous LTC analysis. Identifying the ending clones is a critical point in contig elongation because they have a higher chance than internal clones of overlapping with clones that are not included in the contig. Constructing a consensus band map is more difficult for the contig ends, where the average coverage is lower than in the middle of the contig. In contrast to FPC, the scaffold assembly of LTC does not rely on band map and clone-end coordinates. Instead, identification of contig ends is based on overlaps with MTP clones selected by LTC. Thus, several clones at each of the contig ends are characterized as contig-ending clones [16]. LTC selects the best contig elongations based on the significance of clone overlaps, validated by parallel clone overlaps. If an elongation is not validated by parallel overlaps, LTC selects the most significant clone overlaps. If the difference in significance between the overlap corresponding to ‘the best elongation’ and ‘the next best’ is less than five orders of magnitude, then both variants are considered possible. In such cases, elongation is not performed until additional validating data has been obtained for clarification (for example, molecular markers). A detailed example of contig elongation is presented in Additional file 8.

### Minimal tiling path re-arraying and three-dimensional pooling

MTP re-arraying and three-dimensional pooling were performed as described by Paux *et al*. [22]. Briefly, the 6,447 clones of the MTP were selected from the TaaCsp1BShA BAC library and rearranged into 17 384-well plates containing Luria Broth medium with 12.5 μg/ml chloramphenicol and 6% glycerol. The new coordinates were validated with defined PCR markers. Three-dimensional pools (column pools: 1 to 24; raw pools: A to P; and plate pools: P1 to P17) of the MTP were created and organized into a single 96-well plate that contained 56 DNA samples. DNA was then produced from each pool using the rolling circle amplification method [78] with modifications [22]. Construction of the pools was validated by PCR, as described in [22], with three 1BS-specific markers (Additional file 2).

### BAC-end sequencing

BAC-end sequencing was conducted using 6,447 1BS MTP clones. Sequencing reactions were set up using Big Dye Terminator chemistry (Applied Biosystems, Foster City, CA, USA) according to the manufacturer’s instructions. Both ends of each BAC were sequenced using universal M13 forward (5′-CAGGAAACAGCTATGACC-3′) and reverse (5′-TGTAAAACGACGGCCAGT-3′) primers on a 3730xl DNA Analyzer (Applied Biosystems). Chromatogram traces were base-called and scored for quality using Phred [79].

### Scanning of three-dimensional minimal tiling path pools using the wheat UniGene microarray

The 57 1BS three-dimensional MTP pools were scanned by hybridization with the wheat NimbleGen 40 K UniGene microarray as described in [45]. Briefly, the DNA samples of the 57 MTP three-dimensional pools were sonicated to obtain 500 and 2,000 bp fragments, then labeled using the NimbleGen Dual-Color DNA labeling kit (Roche, NimbleGen) and hybridized with a NimbleGen microarray INRA_GDEC_T.aestivum_NimbleGen_12x40K_unigenes_chip_v2 [80], accession number A-MEXP-2314. Each gene was represented on the chip by three probes marked by two fluorescent dyes. Hybridization and washing of the arrays were performed according to the manufacturer’s procedure (Roche NimbleGen). The arrays were scanned using an InnoScan 900AL scanner (Innopsys). Data were extracted from scanned images using the NimbleScan 2.5 software (Roche NimbleGen). Only UniGenes with unambiguous addresses (plate, row and column coordinates, or those that have coordinates corresponding to overlapping clones) were assigned to 1BS clones and contigs. The strategy used for detecting UniGenes putatively present on 1BS, is described in Additional file 9.

### Construction of 1S GenomeZipper as a reference for anchoring 1BS physical contigs

Chromosome 1BS of wheat is homologous to the *Brachypodium* Bd2 region between genes *Bradi2g30410* through *Bradi2g40150*, rice Os5 between genes *LOC_Os05g01020* through *LOC_Os05g15630* and sorghum Sb9 between genes *Sb09g000200* through *Sb09g008190*[35]. *Brachypodium* contains 600 genes in the 1S syntenic region (after filtering for TE sequences and possible annotation artifacts [35]). To account for genes that might have been moved out of this region in *Brachypodium*, we added (‘zipped in’) those *Brachypodium* genes whose homologs lie in the group 1 syntenic region in both rice and sorghum (as described by [25]). *Brachypodium* contains an inversion between *Bradi2g38650* and *Bradi2g37120* compared to rice Os5 and sorghum Sb9. Thus, for our analysis, we inverted the order of genes between these positions in the *Brachypodium* reference zipper.

### Identification and *in silico* mapping of Illumina sequence contigs

UniGene sequences that gave signals from the hybridized NimbleGen microarray were used for BLASTN searches against the assembly of 1BS Illumina survey sequences obtained from the URGI website [31]. The Illumina sequences that produced hits of at least 200 bp with 90% sequence identity and contained a gene sequence with the closest homolog in the reference 1S GenomeZipper were integrated into the 1BS physical map. Additional sequences were added to the 1BS physical map by BLASTN for all BAC-ends of the anchored contigs. Because most BAC ends are repetitive, only Illumina contigs with a very high similarity of more than 600 bp and at least 99% sequence identity were used.

### Defining 1S GenomeZipper segments corresponding to 1BS deletion bins

The intervals orthologous to 1BS deletion bins [38] were identified on 1S GenomeZipper, using ESTs physically mapped to 1BS deletion bins by RFLP hybridization [39]. The closest homologs of ESTs mapped to deletion bins were found by BLAST (e-value < 10^-10^) on group 1S GenomeZipper and ordered according to their zipper position (Additional file 6).

### Development of PCR-based marker resources

PCR-based markers were developed using 454 sequence data for flow-sorted 1BS arms [35] and BES data for 1BS MTP clones produced in the current study. These 1BS sequences were screened for repeats using cross-match software [81] searching in the TREP databank [82]. The repeats were used to develop primer pairs for ISBP markers using the IsbpFinder Perl script [37]. Primer pairs for SSR markers were developed by the SSRDesign Perl script [37]. In addition, gene-based markers were developed using barley UniGenes [36] that showed synteny with rice Os5 as queries in a BLASTN analysis against the 454 reads of sorted 1BS chromosomes. The reads from 1BS 454 sequences that matched barley UniGenes were assembled separately for each UniGene using Phrap [83]. The assembled 454 contigs were again blasted against the barley UniGenes, and the longest matching fragment was used for primer development. The same procedure was used to develop markers based on *Brachypodium* coding sequences from 1S GenomeZipper [35] (*Brachypodium* coding sequences were obtained from Phytozome [84]). The Primer3 software [85] was used for primer design.

### Plant materials and DNA extraction

Prior to screening the BAC library, the quality of the PCR-based markers (SSR, EST and ISBP) was assessed against the genomic DNA of the reference donor line CS and the nulli-1B-tetrasomic-1A, ditelosomic-1BS and 1BL lines provided by the Wheat Genetics Resource Center, Kansas State University, USA, and against the DNA of flow-sorted 1BS and 1AS chromosome arms [9]. The set of 15 deletion lines of chromosome 1BS was used to assign markers to deletion bins. Plant genomic DNA was extracted using ArchivePure DNA Cell/Tissue MiniKit, (5 PRIME, Hamburg, Germany).

### SSR, ISBP and EST marker analysis

ISBP and EST screening of the 1BS MTP pools and DNA of deletion lines was performed using standard PCR and electrophoresis protocols (Additional file 10). For SSRs, PCR reactions with the M13 protocol [86] were carried out as described in Additional file 10 in a final volume of 15 μl with 200 μM of each deoxyribonucleotide, 500 nM of M13 primer dye-labeled at its 5′-end (Applied Biosystems), 50 nM of the forward M13-tailed primer, 500 nM of the reverse primer, 0.2 U of Taq polymerase (DreamTaq, Fermentas) with 1× of its appropriate buffer and 25 ng of template DNA. PCR amplification was conducted with the touchdown procedure. Amplified products were visualized and analyzed by an ABI 3130xl Genetic Analyzer and the GeneMapper v4.0 software (Applied Biosystems).

### LTC program availability and data deposition

The LTC program may be downloaded from the website of the Institute of Evolution, University of Haifa [17]. The 1BS BAC library is available from the Centre National de Ressources Génomiques Végétales (CNRGV), Toulouse, France [87]. The genome browser of the physical map of the wheat chromosome 1BS and marker data are available from the URGI website [33]. The NimbleGen array design and all microarray data have been deposited with ArrayExpress [80] under accession numbers A-MEXP-2314 for the wheat NimbleGen 40 K UniGene design, and E-MTAB-1912 for the wheat NimbleGen 40 K UniGene hybridization experiment. The Roche 454 sequences of the 1BS sorted chromosome are accessible from the European Bioinformatics Institute short-read archive under the accession number ERP000446. Illumina sequences of wheat sorted chromosomes are available on request from the URGI website [31]. The BES data set is available from The EMBL-European Bioinformatics Institute genome survey sequence under accession numbers HG752437 to HG763829 [88].

## Abbreviations

BAC: bacterial artificial chromosome; BES: BAC-end sequence; bp: base pair; CS: *Triticum aestivum* cv Chinese spring; DAPI: 4',6-diamidino-2-phenylindole; EST: expressed sequence tag; FISH: fluorescent *in situ* hybridization; FPC: FingerPrinted Contigs; Gb: gigabase; HICF: high-information-content fingerprinting; HMW: high molecular weight; ISBP: insertion site-based polymorphism; IWGSC: International Wheat Genome Sequencing Consortium; kb: kilobase; LTC: Linear Topological Contig; Mb: megabase; MTP: minimal tiling path; NCBI: National Center for Biotechnology Information; Nor: nucleolar organizing region; PCR: polymerase chain reaction; Q-clone: questionable clone; Q-overlap: questionable overlap; QTL: quantitative trait locus; RFLP: restriction fragment length polymorphism; RGA: resistance gene analog; R-gene: disease-resistance gene; SSR: simple sequence repeat; TE: transposable element.

## Competing interests

The authors declare that they have no competing interests.

## Authors’ contributions

DR and ZF carried out the physical mapping, the anchoring of the physical map to 1BS deletion bins and 1S GenomeZipper, the comparative genomics and gene space analyses as well as drafting the manuscript. ZF and AK developed the LTC program and implemented it for the scaffolding of BAC contigs. DR, TF and ID produced the PCR marker data. HS developed primer pairs for gene-based markers using 1BS 454 sequences. HŠ and JD sorted the 1BS chromosomes and produced the 1BS BAC library. FM and FC produced the HICF fingerprints of the entire 1BS BAC library and BESs of MTP clones. SV and HB handled the 1BS BAC library and produced the three-dimensional MTP pools. TW and BK produced the GenomeZipper of the wheat chromosome 1BS. PL developed primer pairs for ISBP and SSR markers based on 454 sequences of 1BS. RP, EP, TK and CF conducted the 1BS MTP pool hybridization with the wheat NimbleGen 40 K UniGene microarray and analyzed the hybridization results, and provided Perl scripts for primer pair development. AK and TF acquired the funding, supervised the analyses and drafted the manuscript. All authors read and approved the final manuscript.

## Authors’ information

DR and ZF are the first authors. AK and TF are the senior authors.

## Supplementary Material

Additional file 1 **Chromosome 1BS contigs assembled with FPC and LTC and resulting scaffolds assembled by LTC.** The number of clones per contig and the number of contigs in each category for contigs with 2 to >400 clones obtained by FPC and LTC and classified by size are shown. Click here for file

Additional file 2 **List of 2,939 markers mapped to 1BS BAC clones.** The marker name, the marker type, the name of the deletion bin, the ID of the BAC clones and the name of the physical contigs to which the marker is assigned, for the 2,939 markers assigned to the physical map of the wheat chromosome 1BS, are listed. Click here for file

Additional file 3 **Putative markers developed for 1BS chromosome.** (a) Primer pairs for wheat 1BS chromosome gene-related markers developed based on homology of 1BS sequences with *Brachypodium* Bd2. (b) Primer pairs for wheat 1BS chromosome gene-related markers developed based on homology of 1BS sequences with Barley 1H UniGenes. (c) Primer pairs for ISBP markers developed based on 1BS chromosome 454 sequencing reads. (d) Primer pairs for SSR markers developed based on 1BS chromosome 454 sequencing reads. (e) Primer pairs for ISBP markers developed for 1BS chromosome BES. (f) Primer pairs for SSR markers developed for 1BS chromosome BES. Click here for file

Additional file 4 **List of 1,776 1BS Illumina contigs.** The contigs are in FASTA format. These 1BS Illumina survey sequence contigs [31] were obtained due to their similarity (e-value < 10^-25^) with UniGene sequences that gave signals in the NimbleGen hybridization. Click here for file

Additional file 5 **1S GenomeZipper.** The table shows the homology between *Brachypodium* Bd2, rice Os5 and sorghum Sb9 and markers mapped to wheat chromosome 1BS BAC clones and the established 1BS deletion bins. Gene-derived markers of different types mapped at these positions on clones and contigs with corresponding homologous genes from orthologous regions of the three model grass genomes, *Brachypodium* Bd2, rice Os5, and sorghum Sb9, as well as deletion bin position are presented. Click here for file

Additional file 6 **Delimitation of the syntenic regions of the wheat 1BS chromosome in *Brachypodium *Bd2, rice Os5 and sorghum Sb9.** The text describes how the chromosomal segments corresponding to 1BS chromosome deletion bins were identified using a comparative genomic approach and a table with genes from Bd2, Os5 and Sb9 that delimited the borders of the wheat deletion bins. Click here for file

Additional file 7 **Six major colinearity breaks between 1BS of wheat and Bd2, Os5 and Sb9.** The markers mapped to LTC contigs, their deletion bin position and the corresponding genes from model grass genomes for *Brachypodium*, rice and sorghum are listed. Click here for file

Additional file 8 **Scaffolding by end-to-end merging.** A description of contig elongation by end-to-end merging using the LTC program with figures that illustrate the process. Click here for file

Additional file 9 **Normalization and data deconvolution of the MTP pool data obtained by hybridization with a NimbleGen 40 K UniGene microarray.** A description of the two methods used for normalization and deconvolution of data obtained by hybridization of the 1BS MTP three-dimensional pools with a NimbleGen 40 K UniGene microarray. Click here for file

Additional file 10 **SSR, ISBP and EST marker analysis.** A description of the methods used for PCR reactions. Click here for file
