# Supplementary material for: The physical map of wheat chromosome 1BS provides insights into its gene space organization and evolution
Source: Genome Biol. 2013 Dec 20;14(12):R138. doi: 10.1186/gb-2013-14-12-r138 (PMC4053865; doi:10.1186/gb-2013-14-12-r138)
Supplement: Additional file 1 — Chromosome 1BS contigs assembled with FPC and LTC and resulting scaffolds assembled by LTC. The number of clones per contig and the number of contigs in each category for contigs with 2 to >400 clones obtained by FPC and LTC and classified by size are shown. [file gb-2013-14-12-r138-S1.pdf]

Additional file 1

Table: Chromosome 1BS contigs assembeled with FPC and LTC tools and resulting scaffolds assembled by LTC

	FPC		LTC		LTC Scaffolds	
	clones	contigs	clones	contigs	clones	contigs
Singletons	11894		12777 (including Q-clones)			
Contigs with 2-5 clones	3414	1275	2789	821		
Contigs with >6 clones	34104	518	33846	254	32231	57
Contigs with 6-24 clones	2428	191	782	90	21	2
Contigs with 25-49 clones	3933	112	825	34	64	2
Contigs with 50-99 clones	7902	110	2591	35	353	5
Contigs with 100-199 clones	11097	76	6514	46	1670	10
Contigs with 200-400 clones	6850	26	10478	37	2555	9
Contigs with >400 clones	1894	3	12656	22	27568	29
Total clones in contigs	37518	1793	36635	1075	32231	57
