# Supplementary material for: The physical map of wheat chromosome 1BS provides insights into its gene space organization and evolution
Source: Genome Biol. 2013 Dec 20;14(12):R138. doi: 10.1186/gb-2013-14-12-r138 (PMC4053865; doi:10.1186/gb-2013-14-12-r138)
Supplement: Additional file 6 — Delimitation of the syntenic regions of the wheat 1BS chromosome in Brachypodium Bd2, rice Os5 and sorghum Sb9. The text describes how the chromosomal segments corresponding to 1BS chromosome deletion bins were identified using a comparative genomic approach and a table with genes from Bd2, Os5 and Sb9 that delimited the borders of the wheat deletion bins. [file gb-2013-14-12-r138-S6.pdf]

Additional file 6

Table: Delimitation of the syntenic regions of the wheat 1BS chromosome in *Brachypodium* Bd2, rice Os5 and sorghum Sb9

Wheat 1BS Deletion bins	Established Wheat 1BS Deletion bins	*Brachypodium* Bd2		Rice Os5		Sorghum Sb9	
		from	to	from	to	from	to
1BS.sat18-0.50-1.00*	V	Bradi2g38780	Bradi2g40150	LOC_Os05g01020	LOC_Os05g02130	Sb09g000200	Sb09g001370
1 BS.sat-0.50*	IV	Bradi2g37100*					
1BS9-0.84-1.06*	III	Bradi2g38490	Bradi2g38650*	LOC_Os05g04170	LOC_Os05g04820	Sb09g002640	Sb09g003100
1BS10-0.50-0.84	II	Bradi2g34820	Bradi2g35987	LOC_Os05g05620	LOC_Os05g06690	Sb09g003690	Sb09g004530
C-1BS10-0.50	I	Bradi2g31700	Bradi2g34800	LOC_Os05g06710	LOC_Os05g15630	Sb09g004550	Sb09g008190

*deletion bins partely covered by bins III, IV and V shown in Figure 4.

The intervals orthologous to chromosome 1BS deletion bins [39] were identified on 1S GenomeZipper, using ESTs physically mapped to 1BS deletion bins by RFLP hybridization [40]. The closest homologs of ESTs mapped to deletion bins were found by BLAST (e-value < 10^{-10}) on group 1S GenomeZipper and ordered according to their zipper position. For 20 of the 25 ESTs mapped to deletion bin C-1BS10-0.50 by Peng et al.[40], the closest homologous genes were identified in interval *Bradi2g30460* to *Bradi2g34800* on *Brachypodium* Bd2. The other five ESTs were mapped to three different deletion bins, with three being mapped to the next distal deletion bin 1BS10-0.50-0.84. In the same manner, we found syntenic intervals for C-1BS10-0.50 deletion bin on rice Os5 (from *LOC_Os05g06710* to *LOC_Os05g13520*) and on sorghum Sb9 (from *Sb09g004550* to *Sb09g008430*). Similarly, we defined syntenic intervals for 1BS deletion bins 1BS10-0.50-0.84, 1BS9-0.84-1.06, and 1BS.sat18-0.50-1.00 (Additional files 3 and Table in here). For a chromosome interval corresponding to *Nor* and the proximal part of the 1BS satellite spanning bins 1BS.sat-0.31 and 1BS.sat19-0.31-0.50, it was not possible to identify the bin borders on the model grass genomes due to

poor representation of these bins in the EST mapping study conducted by Peng et al.[40]. Another complication of this comparative genomics approach was caused by the inversion found in Bd2 (*Bradi2g38650* to *Bradi2g37120*), which covers the 1BS chromosomal interval starting from the distal part of the deletion bin 1BS9-0.84-1.06 and ending at the proximal part of the deletion bin 1BS.sat18-0.50-1.00.
