# Supplementary material for: The physical map of wheat chromosome 1BS provides insights into its gene space organization and evolution
Source: Genome Biol. 2013 Dec 20;14(12):R138. doi: 10.1186/gb-2013-14-12-r138 (PMC4053865; doi:10.1186/gb-2013-14-12-r138)
Supplement: Additional file 8 — Scaffolding by end-to-end merging. A description of contig elongation by end-to-end merging using the LTC program with figures that illustrate the process. [file gb-2013-14-12-r138-S8.pdf]

Additional file 8

Scaffolding by end-to-end merging

The correspondence between scaffold names used in the text (and tables) and in the .fpc file with the final version of the assembly: FPC and some of the associated programs do not maintain scaffold structures and textual names for contigs. To overcome this problem, we named scaffolds in *.fpc file (with output of contig/scaffold assemblies) by "ctg" with number. Such names of contigs are suitable for FPC, in the text, we use name "scaffold" with number to differ from the contigs.

Example of contig elongation: We present here an example of scaffolding, starting from LTC based contig Ctg1. By elongation of Ctg1 and stepwise end-to-end merging to it Ctg2, Ctg3 and Ctg5 (but not Ctg4) we obtain a scaffold of 382 clones covering 2.8 Mb of 1BS.

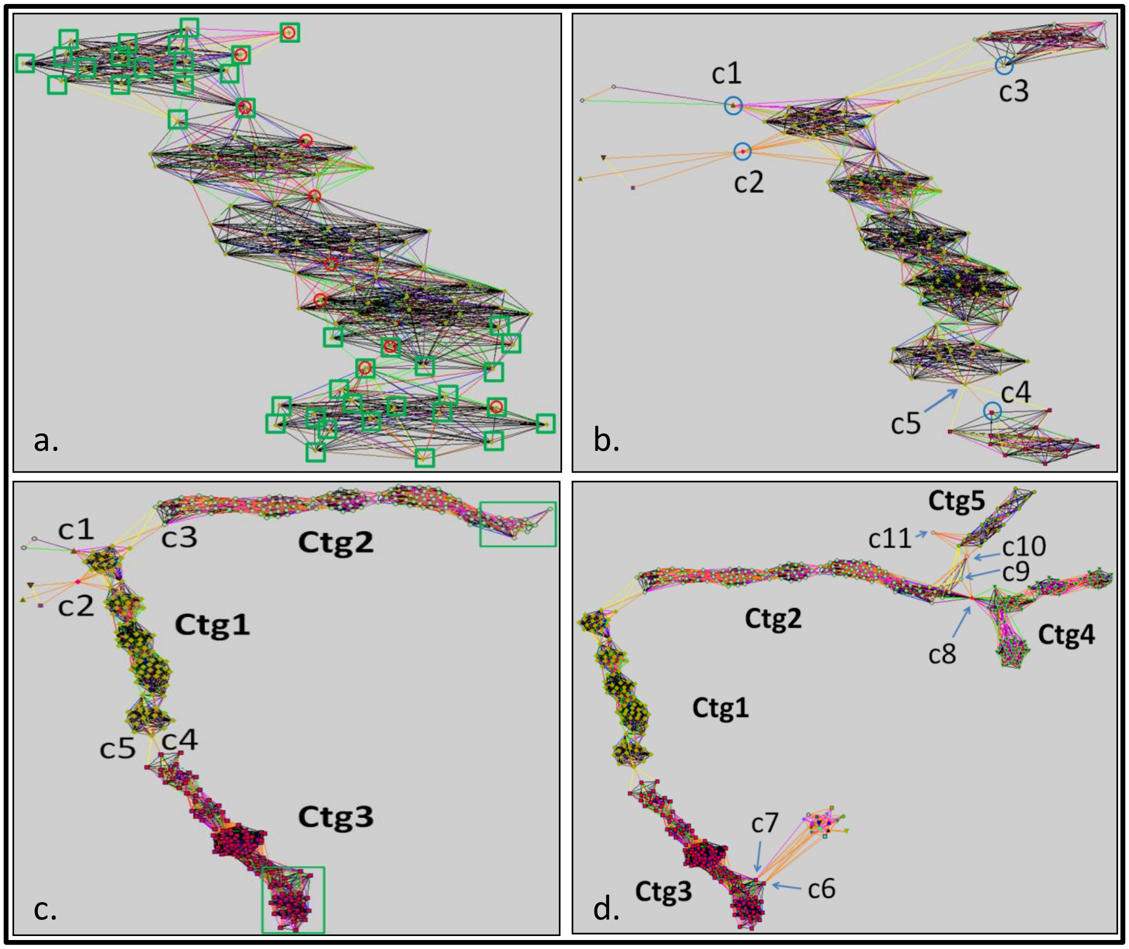

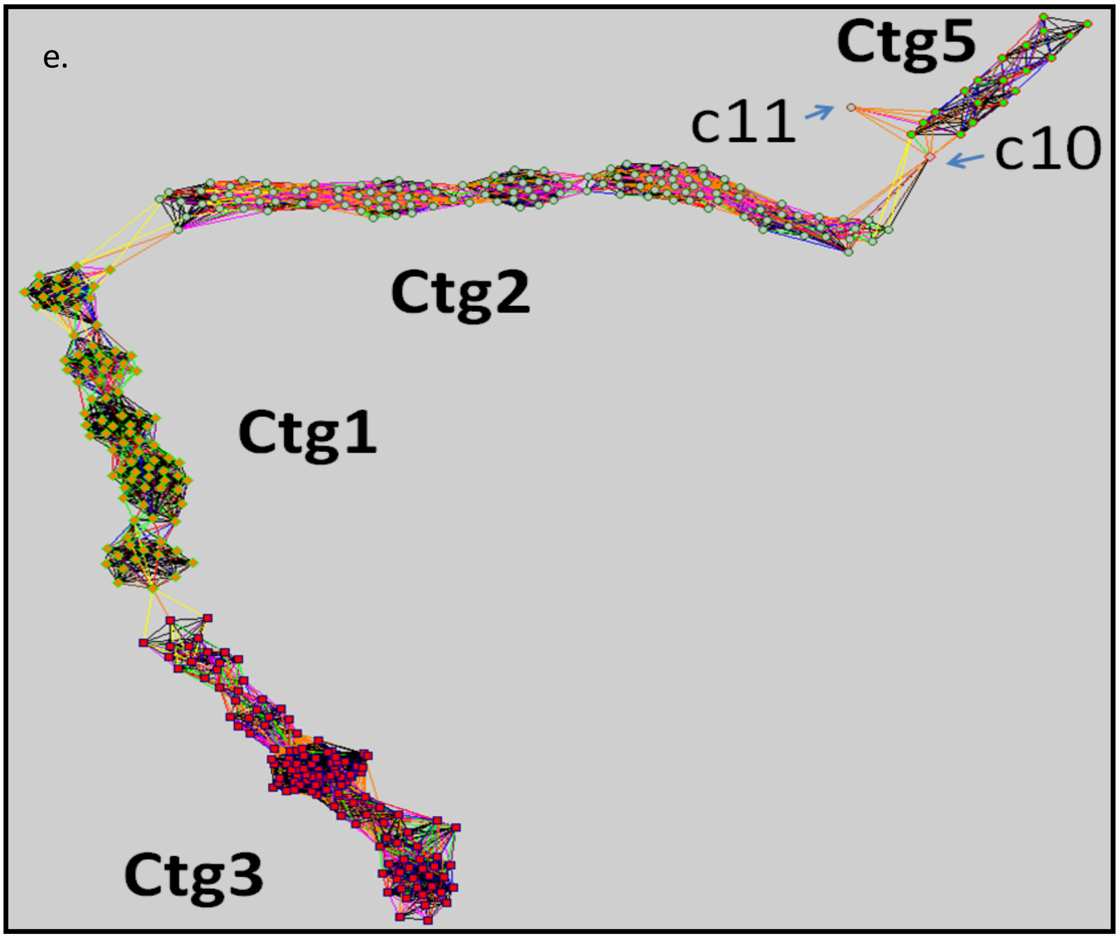

Figure 1. Example of contig elongation and merging. Nodes correspond to clones, edges correspond to clone overlaps. Node shape, color, and color of border are contig-specific. Color of edges reflect Sulston score p-value: yellow means p in the range of 10^{-10} - 10^{-15}, orange – 10^{-15} - 10^{-20}, pink –10^{-20} -10^{-25}, green – 10^{-25}-10^{-30}, red – 10^{-30}-10^{-35}, blue – 10^{-35} -10^{-40}, brown - 10^{-40} - 10^{-45}, and black p <10^{-45}.

(a) Network representation of the initial LTC-based contig Ctg1: Nodes corresponding to clones from diametric path (for details see Frenkel et al. 2010) are marked by red circles while nodes corresponding to contig-ending clones are marked by green boxes.

(b) Elongation of contig Ctg1 via overlaps at cutoff <10^{-15}: Clones having rank=1 are marked by blue circles (yellow edges correspond to overlaps with Sulston score 10^{-10} -10^{-15}). For one end of the contig (top) we have three possible elongation paths: (i) Via clone c1 to pair of overlapped clones having no others overlaps; clone overlaps of clone c1 are unproven by even liberal (10^{-8}) parallel clone overlaps, hence, clone c1 is putatively chimerical; (ii) Via clone c2; clone overlaps of clone c2 are also unproven by liberal (10^{-8}) parallel overlaps. Furthermore, the three linked

clones do not belong to one contig. Such elongation path also seems to go through some internal clones of the contig; (iii) Via clone c3; This variant of contig elongation is proven by parallel clone overlaps (yellow edges). Clones linked with the contig via clone c3 belong to the end of another contigs (Ctg2). Out of these three possibilities, the third one was considered as the best option and it is much better than the two others. For the second contig end (bottom), only one variant of contig elongation was identified (via clone c4). Clones linked with contig via clone c4 belong to the end of another contig (Ctg3), and the overlaps of clone c4 are proven by parallel overlaps (yellow edges). The shortcoming of such elongation is that it is going through clone c5 with overlaps unproven by parallel overlaps (even at liberal cutoff 10^{-8}).

(c) Merging of Ctg1 with contigs Ctg2 and Ctg3: After conducting the steps described above, no other possible elongations were found (at cutoff 10^{-15}) for contigs Ctg2 and Ctg3 from their ends linked to Ctg1 (the opposite ends of these contigs are marked by green boxes). This means that we can allow merging of contigs Ctg1 and Ctg2 via clone c3 (proven by parallel overlaps) and merging of Ctg1 and Ctg3 via clones c4 and c5 (unproven by parallel overlaps). Elongations via clones c1 and c2 should be excluded as less credible.

(d) Further attempts for elongation. Contig Ctg3 can be elongated only via clones c6 and c7. Other clones overlapped with these clones do not belong to a single cluster. Clones c6 and c7 are more “internal” rather than “ending” for contig Ctg3. Overlaps of additional clones with c6 and c7 are unproven by significant overlaps (even at very liberal cutoff 10^{-8}). We consider such contig elongation as problematic and reject it. For contig Ctg2 we found three variants of elongation: (i) via clone c8. Clone c8 is overlapped with the middle of Ctg4. Overlaps of end of Ctg3 and the middle of Ctg4 is unproven by parallel clone overlaps, hence we consider c8 as putatively chimerical and reject such variant of elongation; (ii) Via clone c9. Clone c9 was considered as problematic because it has significant overlaps with clones from a large highly overlapped cluster (not shown); (iii) via clone c10. Clone c10 has an overlap (proven by parallel overlaps, yellow edges) with ending clones of contig Ctg5. End of contig Ctg5 (linked to Ctg2) has an alternative elongation via clone c11 but clone c11 has no other significant overlaps. Therefore, such alternative (elongation Ctg5 by c11) does not contradict to end-to-end merging of contigs Ctg2 and Ctg5.

(e) Resulted net of clone overlaps within scaffold: The foregoing steps result in the assembly of a scaffold Ctg3-Ctg1-Ctg2-c10-c11&Ctg5. The weakest place in the scaffold is the connection between Ctg3 and Ctg1 because it is unproven by parallel clone overlaps.
