# Supplementary material for: The physical map of wheat chromosome 1BS provides insights into its gene space organization and evolution
Source: Genome Biol. 2013 Dec 20;14(12):R138. doi: 10.1186/gb-2013-14-12-r138 (PMC4053865; doi:10.1186/gb-2013-14-12-r138)
Supplement: Additional file 9 — Normalization and data deconvolution of the MTP pool data obtained by hybridization with a NimbleGen 40 K UniGene microarray. A description of the two methods used for normalization and deconvolution of data obtained by hybridization of the 1BS MTP three-dimensional pools with a NimbleGen 40 K UniGene microarray. [file gb-2013-14-12-r138-S9.pdf]

Additional File 9

Normalization and data deconvolution of the MTP pool pata obtained by hybridization with NimbleGen 40K unigene microarray

The 57 1BS 3D MTP pools were scanned by hybridization with wheat (*T.aestivum*) NimbleGen 40K unigene microarray following the protocol described by Rustenholz at al. [1, 2]. Briefly, the DNA samples of the 57 MTP 3D pools were first sonicated to obtain 500 and 2,000 bp fragments, then labeled by the NimbleGen Dual-Color DNA labeling kit (Roche, NimbleGen) and hybridized with the wheat NimbleGen 40K unigene microarray INRA_GDEC_T.aestivum_NimbleGen_12x40K_unigenes_chip_v1 (http://www.ebi.ac.uk/ microarray-as/ae). Each gene was present on the chip by three probes marked by two fluorescent dyes. Hybridization and washing of the arrays were performed according to the manufacturer procedure (Roche NimbleGen). The arrays were scanned using the InnoScan 900AL scanner (Innopsys). Data were extracted from scanned images using NimbleScan 2.5 software (Roche NimbleGen).

Following hybridization, signals were analyzed first to find the MTP addresses of BAC clones carrying the corresponding unigene or at least to detect the unigenes present in 1BS. Data normalization was performed by automated scripts developed with the R software (www.r-project.org) [1, 2]. Data from the MTP pools were made comparable by subtracting the median to each intensity value and then dividing by standard deviation. Two complementary methods were used to define positive clones: (a) the Mean $+\sqrt{\chi^2} \times$ Standard Deviation and (b) Student's *t* test, with three stringency thresholds. The determination of the BAC addresses was carried out independently for both methods and stringency levels by LTC tools. Only unigenes with unambiguous addresses (i.e. having three coordinates: plate, row and column, or having coordinates corresponding to overlapping clones) were assigned to 1BS clones and contigs.

The set of unigenes putatively present on chromosome 1BS was detected using the following criterion (more liberal than one used in the detection of clones carrying unigenes). For each unigene, the MTP pool was considered as a putatively positive if the intensity values of at least two out of the six probe signals representing the unigene on the chip (three probes for each gene scored by two signals) were larger than the predefined threshold value=3.0 (corresponding to 0.001 chance for false positive signal assuming

standard normal distribution under H_0 situation of no hybridization). The proportion of unigenes detected by this criterion was about 0.018 per pool. A unigene was considered as putatively present in 1BS if at least *k* MTP pools (no matter of which type, plate, column, or row) were putatively positive for this unigene (here *k* is the parameter of the criterion). The accuracy of such criterion, even without assuming normal distribution for normalized signal values, can be illustrated by the following calculation. Let $p_1^{(pool)}$ and $p_2^{(pool)}$ be the probabilities to overcome the threshold=3.0 for each of the six signals for any of the unigenes present and absent in the pool, respectively. Let further *h* be the proportion of unigenes present in 1BS. The numerical values of $p_1^{(pool)}$, $p_2^{(pool)}$ and *h* can be estimated by the maximal likelihood method from: (i) the proportions $p_0^{(pool)}$ of probe intensity values (for each pool) ≥3.0 (that was, on average, equal to 0.012 for MTP pool), (ii) proportion of unigenes defined as 'positive' for this pool (equal, on average, 0.018 for MTP pool), and (iii) the proportion of unigenes with maximum intensity value (out of six) ≥3.0 for this pool (on average, 0.031 for the plate pool). On average, we obtained $p_1^{(pool)}$=0.5, $p_2^{(pool)}$=0.002 and h=0.020. Let f_j=1-(1- $p_j^{(pool)})^6$-6 $p_j^{(pool)}$(1- $p_j^{(pool)})^5$, j=0,1,2, be the probability when a pool is considered positive (i.e., at list two of six signal intensities overcome the threshold=3.0). Then, the expected proportion of falsely positive pools for the unigene is only $f = (1- h)\, f_2/(h\, f_1+(1-h)\, f_2)$= 0.003, while the probability to obtain ≥2 falsely positive pools for the unigene is $1-(1-f_0)^{57}-57\, f_0(1-f_0)^{56}$=0.006.

Having the foregoing definition of a positive pool for a given unigene, we need then to decide whether or not this unigene belongs to 1BS. In our analysis, this decision is made by taking into account the information provided by the entire set of 3D MTP pools. The rationale for such joint analysis is that information from single pools may be biased by false positive and false negative detection. Hence joint analysis increases the correct delectability. In an ideal situation, if a unigene belongs to one MTP clone only, we expect to get three positive pools, while for a unigene present in the overlapping part of two neighbor MTP clones, we can obtain up to six positive pools. Depending on the real number of obtained positive pools, we can be more confident or less confident in our statement that the corresponding unigene is present in 1BS. It is easy to show that even if only k=2 out of 57 3D pools of MTP clones were positive, the probability of false positive unigene is equal to $0.5\cdot57\cdot(57-1)\cdot f^2$=0.014.

References

1. Rustenholz C, Hedley PE, Morris J, Choulet F, Feuillet C, Waugh R, Paux E: **Specific patterns of gene space organisation revealed in wheat by using the combination of barley and wheat genomic resources.** *BMC Genomics* 2010, **11**(1)**:**714.

2. Rustenholz C, Choulet F, Laugier C, Šafář J, Šimková H, Doležel J, Magni F, Scalabrin S, Cattonaro F, Vautrin S: **A 3000-loci transcription map of chromosome 3B unravels the structural and functional features of gene islands in hexaploid wheat.** *Plant Physiol* 2011, **157:**1596-1608.
