# Supplementary material for: The physical map of wheat chromosome 1BS provides insights into its gene space organization and evolution
Source: Genome Biol. 2013 Dec 20;14(12):R138. doi: 10.1186/gb-2013-14-12-r138 (PMC4053865; doi:10.1186/gb-2013-14-12-r138)
Supplement: Additional file 10 — SSR, ISBP and EST marker analysis. A description of the methods used for PCR reactions. [file gb-2013-14-12-r138-S10.pdf]

SSR, ISBP and EST marker analysis

ISBP and EST screening of the 1BS MTP pools and DNA of deletion lines was performed using standard PCR and electrophoresis protocols (Additional file 10) by PCR in a 15 μl final volume containing 200 μM of each dNTP, 250 nM of each primer, 0.2 U of Taq polymerase (DreamTaq, Fermentas), and 25 ng of template DNA. PCR was performed on the Veriti 96-Well Fast Thermal Cycler (Applied Biosystems) with the standard PCR program: 5 min of initial denaturation at 94°C followed by 35 cycles at 94°C for 30 sec, 55–60°C (depending on the annealing temperature of the primer set used) for 30 sec and 72°C for 30–60sec (depending on the size of the amplicon) or touch-down PCR program: 10 cycles of 30 sec 95°C, 30 sec 55°C minus 0.5°C each cycle, and 30 sec 72°C, followed by 20 additional cycles of 30 sec 95°C, 30 sec 55°C, and 30 sec 72°C. Amplified products were visualized on 1.5%-agarose gel. For SSRs, PCR reactions with the M13 protocol [86] were carried out in a final volume of 15 μl with 200 μM of each dNTP, 500 nM of M13 primer dye-labeled at its 5′-end (Applied Biosystems), 50 nM of the forward M13-tailed primer, 500 nM of the reverse primer, 0.2 U of Taq polymerase (DreamTaq, Fermentas) with 1X of its appropriate buffer and 25 ng of template DNA. PCR amplification was conducted with the touch-down procedure as described above. Amplified products were visualized with an ABI 3130xl Genetic Analyzer (Applied Biosystems). Fragment sizes were calculated with GeneMapper v4.0 software (Applied Biosystems) where different alleles are represented by different amplification sizes for tandem repeats.
